# Effect of ānāpāna meditation on attention and mental well-being in secondary school students: a mixed-methods evaluation

**DOI:** 10.3389/fpubh.2026.1772248

**Published:** 2026-04-24

**Authors:** Neelam Oswal, Pranita Jagtap, Pradnya Dasarwar

**Affiliations:** 1 Vipassanā Research Institute, Mumbai, India; 2 Jnana Prabodhini’s Institute of Psychology, Pune, India

**Keywords:** ānāpāna meditation, mental well-being, mindfulness, MITRA program, salutogenic process, sustained attention, vipassanā meditation

## Abstract

**Introduction:**

The present study was designed to evaluate the effect of regular ānāpāna practice, a beginning step of vipassanā meditation, in the framework of the MITRA program, over one academic year, on attention and mental well-being among secondary school students.

**Materials and methods:**

Two classes of 8th-grade students from a single school were treated either as experimental group (EG: *n* = 44) or control group (CG: *n* = 45), as suggested by school authorities. MITRA trainers conducted ānāpāna training for students and their class teachers. After this, class teachers conducted regular ānāpāna practice for students for 10 min on every school day using recorded instructions. EG practiced ānāpāna for the entire academic year, whereas CG was waitlisted for the first semester. Both groups were assessed before, mid, and after completion of the program to track changes over time. For students, quantitative measures to assess attention were the figure cancelation task and the digit symbol substitution task, whereas to assess mental well-being, the psychological well-being scale and the NIMHANS sentence completion test (NSCT) were used. Qualitative measures were focus group discussions of students and individual interviews of teachers. Data from parents were taken using a parent rating scale and through individual interviews. A one-way multivariate analysis of variance was conducted to examine and compare changes in the EG and CG over time with respect to the study variables. A deductive approach was used to analyze qualitative data.

**Results:**

At the interim assessment level, gain in adjustment with self, as assessed by NSCT, in EG students was significantly higher than in CG students (Cohen’s *d* = 0.43). There was a significant improvement in sustained attention as assessed by Figure cancelation task of EG students compared to CG by the end of the program (*d* = 0.61). These findings were supported by qualitative data from students, teachers and parents. Qualitative findings clustered around better focus on studies, emotional regulation, positive relations with others, and strategic use of ānāpāna to deal with stress.

**Discussion:**

Based on the present mixed-methods’ findings, it is concluded that regular ānāpāna practice can be useful for educational activities among school-going students (early adolescents). Suggestions are made for better utilization of the salutogenic potential of the MITRA program.

## 1 Introduction

The Mind in Training for Right Awareness (MITRA) program is a joint initiative of the Maharashtra government and Vipassanā Research Institute (VRI) to introduce ānāpāna meditation, a beginning step of vipassanā meditation, to secondary and higher secondary school students (Grade 5th to Grade 10th) since 2011 (1). Details of all government circulars and other details of the MITRA program can be accessed from the VRI website (2). Since then, many school students have been introduced to ānāpāna practice through the MITRA program. To begin this program in a school, trained MITRA volunteers from VRI or local vipassanā centers conduct a training of ānāpāna meditation for students and their class teachers. It is conducted using standard MITRA audio tracks of 70 min. After this training, students practice ānāpāna two times every day, at the beginning and end of the school’s daily schedule. These daily sessions are conducted by their class teachers, playing standard ānāpāna instructions for 10 min. All these are live recordings, in Hindi and English, delivered in the voice of the late principal vipassanā teacher, Satya Narayan Goenka (SN Goenka; 1924–2013), when he offered these instructions to students for the first time. The present study was designed to evaluate the psychological effects of the practice of ānāpāna meditation, introduced through the MITRA program, for students in secondary schools.

The World Health Organization (WHO) defines “adolescents” as individuals in the 10–19 years of age group (3). It can further be divided into early adolescence (10–13 years), middle adolescence (14–16 years) and late adolescence (17–19 years). WHO recommends implementing promotive and preventive interventions for adolescents, as it is a critical developmental stage (4). Internal developmental pressures due to many biological and psychological changes during this period, along with social pressures in the form of competition, social media, and changing morality, are creating more challenges for adolescents these days. Interventions for adolescent mental health can be school-based, community-based, individual/family-based or on digital platforms, as reviewed by Das et al. (5). The interventions may aim to reduce or prevent clinical issues like anxiety, depression, or suicide, or they may aim to improve mental well-being or resilience. The MITRA program fits in the latter category (a well-being intervention) and is closer to the salutogenic model of health promotion as defined by Antonovsky (6). Among the WHO guidelines (4), the MITRA program is closer to the guidelines of creating universal interventions for unselected adolescents.

“Ānāpāna sati” in Buddha’s teachings, generally called as ānāpāna meditation, means developing awareness of ingoing and outgoing breath. With its emphasis on awareness, it is differentiated from other techniques that involve regulation or control of breath. During ānāpāna practice a person is instructed to focus near the nostrils, on the natural breath, and to be aware of it as it is. It provides one-pointedness or concentration (ekaggatā^1^), as well as calmness of mind, which is necessary for the further practice of vipassanā meditation. The objective of vipassanā is to purify the mind by gaining experiential insight into impermanence (anicca), suffering (dukkha), and egolessness (anattā), which are the three essential characteristics of reality (7). Based on its objective, ānāpāna can be considered as concentrative (samatha) meditation, whereas vipassanā is categorized as insight meditation (8). Mettā meditation, which involves developing goodwill toward others, is considered a necessary part of ānāpāna and vipassanā practice and is briefly introduced in the MITRA instructions. Audio tracks of MITRA program show that the focus of daily practice by students in the MITRA program is on ānāpāna meditation (6.5 min). Practice ends with a brief (3.5 min) practice of mettā meditation (9).

Being a concentrative meditation, ānāpāna instructions help a practitioner to develop concentration and also initiate them into mindfulness. While focusing on breath, one is supposed to be vigilant of the wandering mind and keep bringing it back to breath. Due to this component of mindfulness (being aware of reality in the present moment), ānāpāna, or breath awareness, has been incorporated into many mindfulness-based therapeutic interventions right from the seminal mindfulness-based stress reduction (MBSR) (10). In a therapeutic context, mindfulness is defined as having three components (1) awareness, (2) of present experience, (3) with acceptance (11, p. 7). A systematic review of mindfulness-based interventions in the child and adolescent population by Porter et al. (12) shows that formal meditation practices like mindful breathing, which is nothing but ānāpāna meditation, are included in many of these interventions along with various informal mindfulness practices like mindful eating or mindful listening.

Still, the MITRA program is different from other mindfulness-based interventions in teaching only breath awareness and not including any other mindfulness techniques. Awareness of “natural, normal breath without any verbalization, visualization, or imagination” is considered a necessary beginning step for purification of the mind by vipassanā practice (13, pp. 1–3). Proponents of mindfulness-based interventions have often credited the Buddhist origin of these interventions (11, p. 5), but they still missed out on certain aspects from the original context, like morality (sīla). In the MITRA program, ānāpāna practice is introduced as a first step of vipassanā meditation and practice of wholesome behavior (morality) is explained as a necessary foundation for this practice. By incorporating original practice as it is and as the ānāpāna instructions are given directly by a meditation master (SN Goenka), the MITRA program is closer to the original context. Unlike mindfulness interventions, the MITRA program is not a time-bound program and is supposed to become part of the daily school schedule in due course.

A vipassanā course is a systematic teaching of the noble 8-fold path for purifying the mind and the eradication of suffering as taught by Gotama, the Buddha, approximately 2,600 years ago (550 BCE) in India. After resolute working at the mind–body level for 6 years in search of a remedy for human suffering, the Buddha realized and further taught others the four noble truths of human suffering, the noble 8-fold path being one of them (Saṃyutta Nikāya: 56.11) (14). The four noble truths are often explained using a medical model by health care professionals (15). The four noble truths are related to nature, etymology, prognosis, and the treatment of suffering, respectively. Delving deeper into the Buddha’s teachings, it becomes clear that the first two truths are about pathogenesis of human suffering; whereas the latter two truths are about salutogenesis, in the general sense of these two concepts. The present research is more about the salutogenic aspects of Buddha’s teachings.

The noble path leading to the eradication of suffering consists of three divisions: morality, i.e., abstaining from unwholesome deeds of body and speech (sīla), developing mastery over mind (samādhi), and experiential insight into the mind-matter relationship, which totally purifies mind (paññā) (7). Hundreds of years after the passing away of the Buddha, the systematic teaching and practice of the noble path remained with only a few monastics, mostly from outside India. It became available to the masses in the form of 10-day vipassanā courses since 1969, when Acharya SN Goenka, who had learned vipassanā meditation from his Burmese teacher Sayagyi U Ba Khin, started offering 10-day vipassanā courses, first in India and later throughout the world since 1979 (16, p. 87). Vipassanā, being an intense process of purification of the mind, 10-day courses can be availed only by adults. Nonetheless, the lives of children and adolescents are not devoid of stress, more so in the modern competitive world.

It was the great vision of SN Goenka that at least the beginning step of vipassanā meditation, i.e., ānāpāna meditation, be available for children and adolescents before adulthood. The MITRA program is one of the frameworks for introducing ānāpāna meditation to children and adolescents. In this program, ānāpāna is introduced as a skill for gaining mastery over the mind, which is necessary for doing any wholesome action and avoiding unwholesome actions. Abstaining from killing, stealing, misconduct, intoxicants, and harsh speech/lying, all these aspects of morality, are introduced as a necessary foundation for gaining mastery of the mind, which in turn helps in avoiding these unwholesome actions. This training is introduced as a skill which helps for happiness and success in life (9). The objective of the MITRA program is not just to introduce ānāpāna practice and help concentration of mind, but also to introduce students to the path leading to the purification of mind, which they may take up as they grow.

The relation of vipassanā and ānāpāna to salutogenesis has been pointed out, time and again, by meditation practitioners and researchers, although this ancient technique is not necessarily for health promotion. Salutogenesis is referred here in its most general meaning, i.e., “a salutogenic orientation particularly in health promotion research and practice, is focusing attention on the health and assets for positive health, contra to the origins of disease and risk factors” (17, p. 17). Fleischman (18, pp. 58–61), a psychiatrist and vipassanā practitioner, pointed out that though according to Buddha’s teachings vipassanā meditation is to be practiced for realization of nibbāṇa, i.e., complete eradication of all defilements (7), as one starts practicing and advances in it, many health benefits at molecular (alteration in the flow of stress hormones), biological (changes in weight, heart rate, alertness, dietary choices, sleep patterns), psychological (old complexes abandoned, positive attitudes learnt), inter-personal (new ways of interacting with people), and ecological levels (respect for all living beings) are experienced.

In the last 50 years, as vipassanā courses are being offered regularly all over the world, the impact of vipassanā on health and well-being has been researched empirically time and again and confirmed psychological and physiological benefits of vipassanā meditation (19, 20). Health benefits of vipassanā meditation are researched as operating through psychological mechanisms like metacognition, observing thoughts without attachment, and fostering emotional regulation and resilience against negative affect, as well as some neural mechanisms (20). Kabat-Zinn (10, p. 249) repeatedly observed increased stress hardiness and sense of coherence (SOC) among participants after completing the MBSR program of 8 weeks, in which breath awareness is introduced along with body scan, sound awareness, awareness of thoughts and feelings, mindful yoga, walking meditation and mindfulness in daily life. Similar results of increased SOC were observed in a group of women with fibromyalgia after participation in the MBSR program in comparison to wait-listed controls (21).

Although there is empirical evidence for the health-promoting aspects of vipassanā as well as of ānāpāna, Buddha’s teachings suggest that the salutogenic process started by the practice of vipassanā can be much deeper than that of the practice of ānāpāna or mindful breathing, as adopted in mindfulness-based interventions. To appreciate the difference between the salutogenic process initiated by vipassanā and ānāpāna, we need to understand the concept of mind and the elaboration of the processes of vipassanā in Buddha’s teachings.

A person keeps coming in touch with either external (sight, sound, smell, taste, and touch) or internal (thoughts/images) stimuli every moment. Every stimulus sets in motion four mental processes (khandha). They are: consciousness (viññāṇa) of a stimulus at any of the sense doors, perception and evaluation of the stimulus (saññā), arising of pleasant, unpleasant, and neutral bodily sensations (vedanā), and finally, reaction (saṅkhāra) of liking (rāga) or disliking (dosa) the stimulus (22). These momentary liking and disliking lead to great craving and a strong attachment to the stimuli producing these sensations. One wants to have those stimuli repeatedly that trigger pleasant sensations and feels repulsive against stimuli that trigger unpleasant sensations. Because of the attachment one suffers when one does not get what one wants, or comes across something not of his/her liking (Dīgha Nikāya: 15) (23).

In vipassanā meditation, one is trained to be aware of subtle-most bodily sensations, observe their impermanent nature, and not give the usual reaction of liking or disliking for the uprooting of suffering. Instead, one is trained to be equanimous based on direct experience of their changing nature. Thus, developing awareness/mindfulness (sati) and equanimity (upekkha) are two aspects of this technique (13, p. 34). The experientially learnt equanimity during a vipassanā course is supposed to reflect in daily life full of complexity and chaos. Like every stimulus, a stressor also gives rise to pleasant and unpleasant sensations as soon as one becomes aware of the possibly stressful situation, even before any appraisal is made. In the face of a stressor, a person who is practicing vipassanā regularly becomes aware of his/her bodily sensations either intentionally or sometimes unintentionally as well, which are an early sign of a stress reaction. This grounding in the present moment bodily sensations creates a pause to the mental process, which has probably started rolling into automatic negative thoughts.

The importance of grounding in the present moment for changing the automatic reaction pattern in a stressful situation has been discussed by mindfulness-based practitioners as well (10, p. 336). However, the key salutogenic mechanism of vipassanā is much more than just grounding in bodily sensations, or being in the present moment. It is the direct experience of impermanence at the level of bodily sensations, and equanimity based on this experience (7, p. 37). In the instructions of ānāpāna meditation, there are elements of non-judgmental awareness or acceptance, but they may not facilitate the experience of impermanence in a beginning practitioner. Despite this difference in the salutogenic process initiated by ānāpāna and vipassanā meditation, can practice of ānāpāna contribute to mental well-being is a background question for the present research.

The well-being framework and research by Dahl et al. (24) answer this question affirmatively. Awareness, a heightened and flexible attentiveness to perceptual impressions in one’s environment, as well as internal cues, such as to bodily sensations, thoughts, and emotions, is an important component of the well-being framework as suggested by Dahl et al. Other components in this framework are connection, insight, and purpose. In a large-scale study (25) of more than 5,000 people from 83 countries, it was observed that people are in a state of distraction almost 50% of their waking time. Distraction and its converse, awareness, are directly related to their well-being. Here, awareness is like a meta-awareness wherein one becomes aware as one loses attention while doing any activity. Dahl et al. have listed breath awareness as a traditional contemplative intervention which can enhance awareness, a component of well-being.

In the present research, the answer to the above question is explored in a group of adolescents who practiced ānāpāna meditation in the framework of the MITRA program. Though thousands of students in Maharashtra have benefited from school-based teaching of ānāpāna meditation through the MITRA program since 2011, there has been little empirical evidence for the reported benefits so far. Anecdotal evidence of the benefits of the MITRA program, narrated by students and school teachers, was recorded in a documentary film in 2017 (26). Empirical evidence may help improve and further the outreach of the MITRA program.

There are many studies wherein the effect of mindfulness practices for non-clinical populations among children and adolescents has been assessed (12). Mindfulness practices in these interventions were both formal and informal. Formal practices, too, were a mix of mindful breathing, yoga, and body scan. Informal practices were mindful listening, mindful eating, meditation bubble, etc. Outcome variables in these studies varied and were related to behavior, emotional functioning, well-being, anxiety, self-regulation, mindfulness, perceived stress, and social contact. However, mindfulness interventions being a mix of many techniques, it is difficult to isolate and differentiate the effects of any technique on these variables from others. In the MITRA program, participants have practiced only breath awareness (ānāpāna), with a brief practice in developing goodwill toward others (mettā) at the end. Hence, the present research would be helpful to identify the core effect of breath awareness.

There have been few studies assessing the effect of ānāpāna practice among adolescents. Bhutekar and Shirsath (27) have tried to assess the effect of ānāpāna practice on stress and learning ability among 60 students in the age range of 12–16 years, selected by a purposive sampling method, in Ambad city of Maharashtra. It was concluded in this study with a pre-post research design that ānāpāna practice is useful to reduce stress and increase learning ability among adolescents. Pandey (28) has chosen 240 students from two secondary schools in Kathmandu, Nepal, who have taken ānāpāna courses and continued their practice. A structured questionnaire set was designed to seek first-hand information from these students about nine different clusters of behavior. Students reported that regular practice of ānāpāna improved their study habits and academic performance. In addition to that, regular ānāpāna practice was helpful to improve physical as well as mental health, reduce anger, and improve relationships with friends, family members, and their relatives.

Studies on ānāpāna show that while assessing the effect of ānāpāna among secondary school students, academic performance seems to be the natural choice of researchers. Nonetheless, the film on MITRA (26) as well as the government resolution through which the MITRA program was initiated in 2011 (1) have mentioned four different categories of benefits of ānāpāna practice for students. They are improvements in cognitive variables (attention, concentration, and memory); more positive emotions and behavior (confidence, happiness, and affectionate behavior toward friends); a decrease in negative emotions (fear, anger, depression, and stress); and better academic performance. It is felt necessary to assess all these categories of benefits together to understand the interrelation among them after ānāpāna practice. In the present study, the first category of benefits is addressed as “attention,” whereas the second and third categories together are addressed as “mental well-being.”

In a critical systematic review of interventions to enhance sustained attention among children and adolescents (3–18 years), Slattery et al. (29) have identified three categories of interventions: cognitive attention training, meditation training, and physical activity interventions. They have found that mindfulness training has a consistent positive effect on selective attention and has more potential for enhancing sustained attention. Based on this review, it is hypothesized in the present research that concentration and mindfulness, developed by ānāpāna practice, would enhance performance on tasks requiring two facets of attention: sustained attention and working memory. Self-regulation, which is required in ānāpāna practice for bringing wandered mind back to the breath repeatedly, is presumed to lead to mental well-being based on a systematic review of school-based mental well-being interventions among adolescents by Cilar et al. (30). Inclusion of a brief practice of mettā while ending ānāpāna is thought to contribute to some aspects of mental well-being, like inter-personal relations. Thus, attention and mental well-being are treated as dependent variables (DVs) while evaluating the psychological effects of the practice of ānāpāna meditation in the present research. They are assessed using quantitative as well as qualitative methods.

Cilar et al. (30) have systematically reviewed and synthesized evidence on school-based interventions aimed at promoting mental health and well-being among adolescents (10–19 years old). Different types of school-based interventions among the 57 reviewed studies can be categorized under three themes: psychological well-being (quality of life, mental well-being, anxiety and depression, coping, and personal growth), subjective well-being (positive psychology, emotional well-being, social and emotional skills, and bullying prevention), and psychosocial well-being (social inclusion, peer relationships, mindfulness, physical activity, and stress management). Those based on positive psychology, mindfulness, and programs integrating physical activity and social skills were found to be the most effective ones in this review, though not all mindfulness-based interventions were highly effective. Most of these studies have used quantitative measures to evaluate the intervention. Looking at the inconsistent results of these studies, it was felt necessary to use mixed methods for a thorough evaluation of the effects of ānāpāna. The need for diverse assessment strategies is emphasized even by Porter et al. (12) while evaluating mindfulness-based interventions for youths. Feedback from teachers and parents who observe students closely was felt to be necessary for the triangulation of qualitative findings from student participants.

Sustained attention, commonly called as concentration or vigilance, is the ability to focus on one object for a required duration of time. Tasks measuring this ability require the detection of simple stimuli, presented infrequently in the stream of other stimuli. Working memory is the ability to hold information in consciousness for adaptive use. It provides temporary storage (maintenance) and manipulation of information necessary for complex cognitive tasks (31). In the present research, sustained attention is measured using the figure cancelation task (FCT), and working memory is measured using the digit symbol substitution task (DSST).

Mental well-being is an important component in the definition of mental health by the World Health Organization (WHO). As defined by WHO, mental health is a “state of well-being in which an individual realizes his/her own abilities, can cope with normal stresses of life, can work productively and fruitfully and is able to make contribution to his/her community” (32). Westerhof and Keyes (33) separated emotional, psychological, and social well-being in this definition. Emotional well-being refers to feelings of happiness and satisfaction. Psychological well-being refers to positive psychological functioning in terms of self-realization. Social well-being refers to positive societal functioning in terms of being of social value. In the present research, the psychological well-being of the participants is assessed using a rating scale based on the psychological well-being model by Ryff (34). The six dimensions of psychological well-being in this model are as follows:

1. Self-acceptance: a positive and accepting attitude toward aspects of self in the past and present;
2. Purpose in life: goals and beliefs that affirm a sense of direction and meaning in life;
3. Autonomy: self-direction as guided by one’s own socially accepted internal standards;
4. Positive relations with others: having satisfying personal relationships in which empathy and intimacy are expressed;
5. Environmental mastery: the capability to manage the complex environment according to one’s own needs;
6. Personal growth: the insight into one’s own potential for self-development.

A school-going adolescent’s social environment mainly consists of relations with peers (in and outside school), siblings, parents, teachers, and the school system. Better psychological well-being is expected to result in better adjustment at home, at school and with self. In the present research, a semi-projective test, the NIMHANS sentence completion test (35, 36), is used to assess participants’ adjustment to self and others. Findings in some of the areas in this test may overlap with the psychological well-being test. Still, it is chosen as an adjunct to the psychological well-being test to reduce possible social desirability in a self-report inventory and increase the possibility of getting a more authentic picture of mental well-being. Apart from psychological tests, the effect of ānāpāna on DVs is explored by analyzing students’ narrations in focus group discussions (FGDs) and teachers’ and parents’ narrations in individual interviews.

Thus, the objectives in the present research are:

1. To understand the effects of regular ānāpāna practice over one academic year on two facets of attention, namely, sustained attention and working memory, among secondary school students.
2. To understand the effects of regular ānāpāna practice over one academic year on two facets of mental well-being, namely, psychological well-being and adjustment to self and others.
3. To understand the trajectory of changes in these study variables over one academic year.

It is hypothesized that ānāpāna practice will have a positive effect on all the four study variables.

## 2 Materials and methods

A quasi-experimental two-group, experimental and control, with repeated measures research design was used for evaluating the effect of ānāpāna practice on the DVs in the present study. Both groups were assessed before (pre), mid (interim), and after completion of the program (post) to track changes over time.

### 2.1 Participants

#### 2.1.1 Students

Participants were 8th-grade students from an English-medium, co-education, private school, with state board curriculum in Pune, a city in the Maharashtra state, India. Out of the four classes of 8th grade in this school, two classes were allotted by the school authorities for this research. Of these two classes, one entire class was treated as the experimental group (EG) (*n* = 44), and the other entire class was treated as the control group (CG) (*n* = 45), as suggested by the school authorities. Most of the participants were from the middle or higher middle socioeconomic class, except for a few who were given admission under Right to Education (RTE) quota as per the education policy^2^ (37). Demographic details of student participants are given in Table 1.

**Table 1:** Demographic details of students.

Variables	Experimental group	Control group
Total	44	45
Girls	19 (43%)	22 (49%)
Boys	25 (57%)	23 (51%)
Mean age (in years)	13.16	13.19
Number of students in RTE category	9 (20%)	12 (27%)

RTE, right-to-education.

#### 2.1.2 Teachers

Teachers teaching the EG (*n* = 6) and the CG (*n* = 5) were enrolled as respondents to understand the effect of ānāpāna practice on the classroom behavior of students. Teachers enrolled from EG were the class teacher (also teaching science and mathematics), two language teachers (English and Sanskrit), social science teacher, sports teacher, and arts-crafts teacher. Teachers enrolled from CG were the class teacher (also teaching mathematics), science teacher, language teacher (Marathi), sports teacher, and arts-crafts teacher.

#### 2.1.3 Parents

Parents of students in the EG and CG were enrolled as respondents to understand changes observed by them in their children’s behavior. Parents who were present for the parents-teacher meeting or were ready to give time were enrolled as respondents.

Inclusion and exclusion criteria for students, teachers, and parents are listed in Table 2.

**Table 2:** Inclusion and exclusion criteria for participants.

Eligibility criteria	Students	Teachers	Parents
Inclusion criteria	Students in the 8th grade Classes of 8th grade, with permission for research enrolment by school authorities Consent given by parents	Subject teachers with regular interaction with students in the year 2024–2025 Class teachers of each class Teachers teaching science/mathematics, one of the languages, sports, and art-craft	Parents who were present for parent-teacher meeting Ready to give time for individual interviews
Exclusion criteria	Students of 5th to 7th grades and 9th and 10th grade Students for whom consent was not given by parents	Teachers who are not teaching the participant students	Parents who were not present for parent-teacher meetings Not ready to give time for individual interviews

### 2.2 Intervention

Intervention in this research was the MITRA program designed by VRI (2), spanning over an entire academic year. As per the standard design of the MITRA program, intervention was carried out in three steps:

- A MITRA orientation session: Introduction of vipassanā and ānāpāna meditation for all the teachers and willing parents by volunteers from Pune vipassanā center in the beginning of the first semester.
- MITRA training: Training of ānāpāna meditation for participant students and their class teachers by trained MITRA volunteers from Pune vipassanā center using recorded (Hindi) audio tracks of the MITRA program of 70 min. Revision of the same training was done at the beginning of the second semester.
- Daily practice of ānāpāna meditation for 10 min in school by the student participants: Instead of joining the common school assembly, students practiced ānāpāna in their respective classrooms. These daily sessions were conducted by their respective class teachers playing recorded (Hindi) instructions of ānāpāna.

Details of these steps are given in Table 3. In the MITRA program, students are supposed to practice ānāpāna two times every day, once as school begins, and a second time as school ends. However, due to the unavailability of time in the school schedule, the second time practice was left to be done at home. Parents were provided a link to recorded ānāpāna instructions for the same.

**Table 3:** Three steps of the MITRA program.

Details	Step 1: MITRA orientation	Step 2: MITRA training	Step 3: Daily ānāpāna practice
For whom?	Interested teachers and parents of students in a school	Students and their class teachers	Students
By whom?	MITRA volunteers from VRI/ local vipassanā center	MITRA volunteers from VRI/ local vipassanā center	The class-teacher who has received MITRA training
Contents of the session	Introduction to vipassanā (ITV), information on MITRA, and practice of ānāpāna	Introduction to ānāpāna as a technique to control the mind, for living a peaceful life. Instructions to practice ānāpāna. Possible obstacles and ways to overcome them. Practicing metta	Practice of ānāpāna meditation
How?	Using ITV material (50–60 min)	4 audio tracks of the MITRA program: Track 1–16 min Track 2–13 min Track 3–15 min Track 4–20 min	Recorded instructions for ānāpāna meditation (10 min)
Frequency	One time a year	At the beginning of every semester	Every day, two times for 10 min*

"*" is the standard of ānāpāna practice in the MITRA program. It had to be adapted to once a day in the present research.

### 2.3 Measures

#### 2.3.1 Quantitative

##### 2.3.1.1 Ānāpāna practice record

The number of days when ānāpāna was practiced by each student in school was recorded manually by the respective class teachers. Second time practice at home was encouraged by these class teachers. However, ānāpāna practice at home was not recorded.

##### 2.3.1.2 Figure cancelation task

The Figure cancellation task (FCT) measures sustained attention that combines both visual selectivity and motor response. The test consists of a sheet of various figures randomly arranged. Two figures are given as target figures. Students are given the choice of two possible strategies to cancel the target figure in the test sheet. Students can cancel two figures at one time or select a single target figure at a time. According to their own choice, they follow horizontal, vertical, or random paths on the task sheet. They are asked to cancel as many target figures as possible in the given time of 1 min. The score is the number of correct cancelations of figures. Standard instructions for all quantitative measures were given in English. In case the English instructions were not clear, instructions were repeated in Marathi.

##### 2.3.1.3 Digit symbol substitution task

The Digit symbol substitution task (DSST) is a paper-and-pencil task to assess working memory. Participants are given a key grid of numbers and matching symbols. In the task section on the same sheet, numbers and empty boxes are given. The task consists of filling as many empty boxes as possible with a symbol matching each number. The score is the number of correct number-symbol matches achieved in 90 s. This task is taken from the Indian adaptation of the Wechsler performance intelligence test, developed by Prabha Ramalingaswami (38).

##### 2.3.1.4 Psychological well-being scale for adolescents

The psychological well-being scale was developed by Kulkarni (39) for adolescents. This scale is based on Ryff’s model of psychological well-being (34). It consists of six dimensions: self-acceptance, purpose of life, autonomy, positive relations with others, environmental mastery, and personal growth. This is a 4-point Likert scale and consists of 61 items. Test items are available in English as well as Marathi. The test was administered in English. Students could ask the meaning of a word, in case they did not understand. Marathi translation of the test items was referred to while answering any query. The test-taker chooses one from the four choices given: “totally agree,” “agree,” “somewhat disagree,” “totally disagree,” and ticks in the appropriate box across each statement. Possible score range is 61–244, with 244 indicating optimal functioning and 61 indicating struggling of an adolescent across the six dimensions of psychological well-being. Cronbach’s α coefficient for the total score is 0.88, and for subscales, it ranges from 0.56 to 0.67. Test–retest reliability is 0.79 after a 3-month gap. A content validity is also established by five experts. This scale is moderately negatively correlated (*r* = −0.53) with the child and adolescent scale of irrationality.

##### 2.3.1.5 NIMHANS sentence completion test

The NIMHANS sentence completion test (NSCT) for children and adolescents is a standardized semi-projective test for children and adolescents in the Indian setting (35). The test assesses a child’s perceptions, attitudes, and interactions to arrive at a comprehensive understanding of the child’s adjustment. The test covers four main domains, such as home, school, self, and friends. Test items are 75 incomplete sentences. The test taker is to complete an incomplete sentence as per his/her perception. Clinical validity is established by comparing scores on this test of children with psychopathology and without any psychopathological symptoms (36). The number of items was reduced to 55 in consultation with the test authors for the present purpose, due to the limited time available in a school setup, while retaining the same domains and subdomains. The original English test was translated into the Marathi language for the present study. Test items were presented simultaneously in English and Marathi on the test sheet. Students were instructed to choose the language of their convenience while completing the sentences. A person’s responses on NSCT are to be scored by experts in mental health as 0, 1, and 2. Score 0 indicates good adjustment, 1 indicates mild problems in adjustment, and 2 indicates significant problems in adjustment. Excluding the two neutral items in the adapted version, scores range from 0 to 106, with lower scores indicating better adjustment.

##### 2.3.1.6 Parent rating scale

The parent rating scale everywhere was developed by Kulkarni (39). It assesses parents’ perception of the psychological well-being of their children. It has 21 items. Test items are available in English and Marathi. Parent rating scale test items were presented simultaneously in English and Marathi on the test sheet. Parents were instructed to choose the language of their convenience. The test taker is to respond on a 5-point rating scale ranging from always to not at all. Scores range from 21 to 105. Higher scores indicate optimal functioning of the child, as observed by his/her parents, across the six dimensions of psychological well-being of Ryff’s model (34). Cronbach’s alpha of this scale is 0.79.

#### 2.3.2 Qualitative

##### 2.3.2.1 Focus group discussion

Focus group discussions (FGD) of students from the same class were conducted on a single day by trained facilitators and co-facilitators in small groups of eight to nine students. In each class, five groups were made for the purpose of FGD. One was an all-girls’ group, one was an all-boys’ group, and three were mixed groups. Care was taken that there was an equal number of girls and boys in the three mixed groups. Students in each class were assigned randomly to the five groups. A researcher-designed FGD schedule was used to facilitate the group discussion. Objectives of the FGD were to understand participants’ perceptions and opinions about ānāpāna practice and the MITRA program, and to understand their perception about the effect of ānāpāna on their academic and non-academic performance, self-perception, and personal and social relations. All the FGDs were conducted in the Marathi language, the mother tongue of almost all participants. The average duration of each FGD was 60 min. All FGDs were audio-recorded with the consent of participants.

##### 2.3.2.2 Individual interviews with teachers

Class teachers who were conducting ānāpāna sessions for students every day and subject teachers who observed students regularly during the intervention period were interviewed by trained interviewers. Interviews were conducted in the school premises on a single day, except for two, which had to be conducted in online mode due to the unavailability of these teachers in person on the pre-decided day. A researcher-designed interview schedule was used for the same. Objectives of teachers’ interviews were to understand teachers’ observations about the effect of ānāpāna practice on classroom discipline, students’ attention and comprehension level, their attitude toward assessment, inter-personal relations, and performance in sports and extra-curricular activities. Teachers responded mostly in the Marathi language. The average duration for each interview was 60 min. All the interviews were audio-recorded with the consent of teacher respondents.

##### 2.3.2.3 Individual interviews with parents

Totally, 20 parents from both the groups were shortlisted for individual interviews based on their child’s general classroom behavior as suggested by the respective class teachers. From the shortlisted parents, those who agreed (*n* = 5) were interviewed by trained interviewers. All the parents were interviewed telephonically. The objective was to understand what changes parents noticed in their children as they started practicing ānāpāna. A researcher-designed interview schedule was used for these interviews. Marathi was the language of comfort for most of the parents during these interviews. The duration of parents’ interviews ranged from 30 to 60 min.

### 2.4 Ethics approval

Approval from the Institutional Ethics Committee (IEC) was obtained for this study (JPSS-IEC/July2024/Project-5). After getting IEC approval, permission from the school authority, and informed consent from students’ parents were obtained for the research process.

### 2.5 Procedure

Pre-assessment of both the groups on the selected quantitative measures was done in the beginning of the academic year 2024-25. EG students were trained and started practicing ānāpāna meditation immediately after their pre-assessment. Parents of both the classes were administered the parents’ rating scale during their parent-teacher meeting after the MITRA orientation session. An interim assessment of students in both the classes was done at the end of the first semester in October 2024. The gap between pre and interim assessment was 89 days. Five FGDs of EG students were conducted at the interim assessment level. Teachers of EG were interviewed at this point.

Students in CG who were waitlisted in the first semester were trained and started practicing ānāpāna meditation immediately after 15 days of the mid-semester school vacation, following the interim assessment. Students in EG went through a revision of ānāpāna training at this time. Post-assessment of students in both the classes was done toward the end of the second semester, in February 2025. The gap between the interim and post-assessment was 90 days.

Five FGDs of students in each group (EG and CG) were conducted at the post-assessment level. Teachers of both the classes were interviewed. Parents from both the classes took the parents’ rating scale again this time, in a parent-teacher meeting at the school. Parents were interviewed at the end of the program.

**Figure 1:**
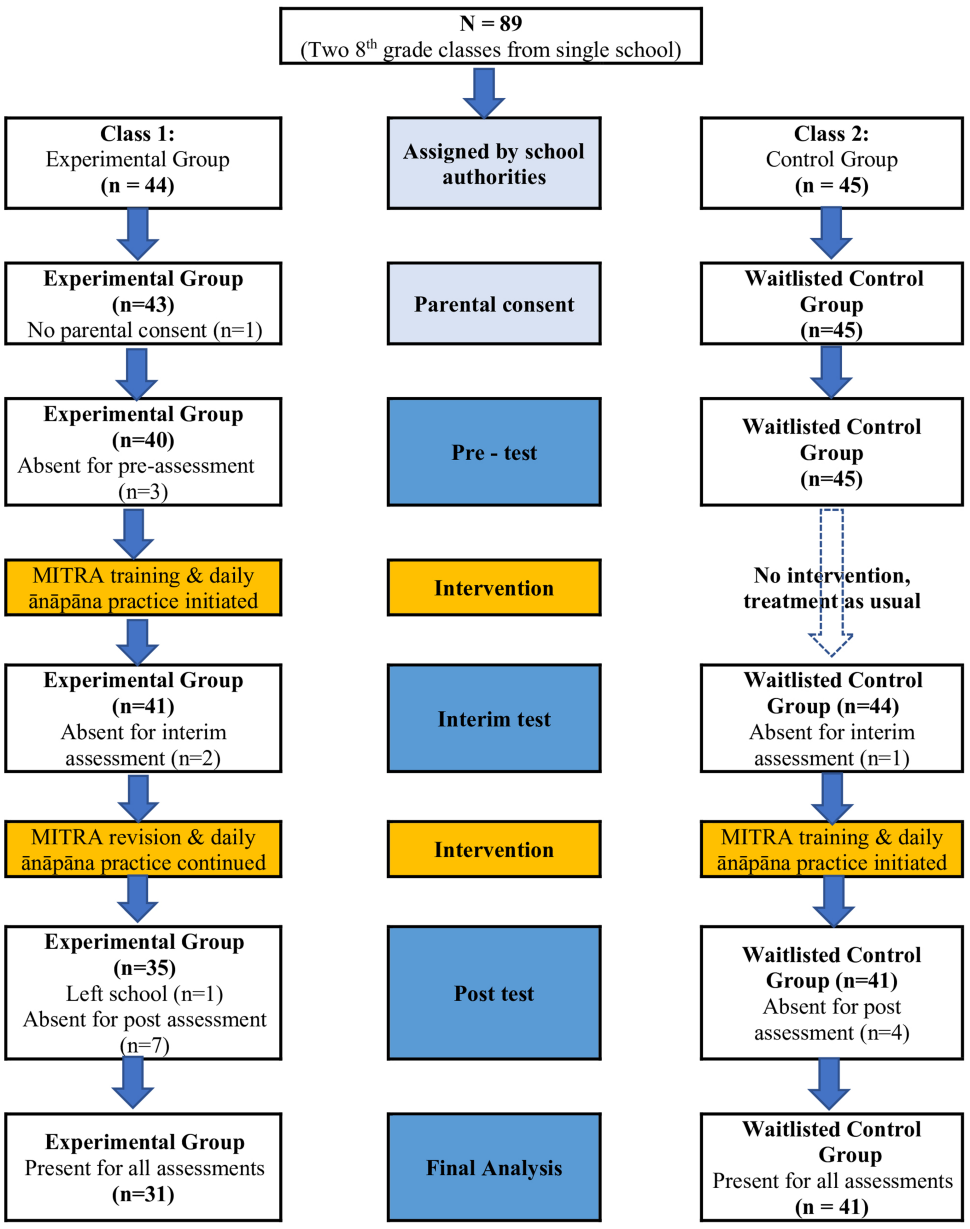
The research procedure.

The number of students in each group, at each stage of the research, right from assignment of the entire class to experimental or working memory till final analysis, along with reasons for data attrition, are shown in Figure 1.

### 2.6 Analysis

#### 2.6.1 Statistical analysis

##### 2.6.1.1 Students

Quantitative data from students were scores on all four tests, at three times (pre-post-interim), of both the groups, experimental (EG), and control (CG). A pre-test was conducted on data from 88 students, and a final analysis was run on data from 72 students. Students who were present for all three test times were included in the final analysis, irrespective of the number of ānāpāna practice sessions attended by them. A one-way multivariate analysis of variance (MANOVA) followed by a univariate test was conducted to examine and compare changes between the EG and CG over time in study variables.

Before MANOVA, equivalence of the EG and CG was checked using an independent *t*-test on their pre-test scores. No significant differences in pre-test scores showed that both groups were equivalent, except for working memory (Supplementary Table S1).

Normality of the study variables was assessed using the Shapiro–Wilk test, which indicated that both groups were normally distributed, except for adjustment of CG (Supplementary Table S2).

Box’s M test for homogeneity of variance showed that both groups have equal variances on sustained attention and working memory. However, this test could not be computed for other study variables because fewer than two non-singular covariance matrices were available. Hence, homogeneity of variances for other DVs was checked using Levene’s test (Supplementary Table S3). As the *p*-value was greater than 0.01 for both the tests, the assumption of homogeneity of variances was satisfied.

Correlations of all DVs were also checked, calculating Pearson’s correlation (Supplementary Table S4). It ranged from *r* = 0.016 to *r* = −0.231, indicating low-to-moderate relationships, which suggests that the assumption of multicollinearity was fulfilled.

Three separate MANOVA (sustained attention and working memory together, psychological well-being, and adjustment) were calculated on gain scores. Gain-1 (interim test scores minus pre-test scores) and Gain-2 (post-test scores minus interim test scores) were used to compare group differences, and Pillai’s trace was used to interpret the MANOVA. Following this, univariate analysis was calculated to examine changes between the EG and CG over time in study variables.

Descriptive statistics of all study variables for students at three time-points (pre, interim, and post-test) and Gain-1, Gain-2 are given in Table 4.

**Table 4:** Descriptive statistics of all study variables for students (mean ± standard deviation).

	Experimental group (*n* = 31)					Waitlisted control group (*n* = 41)				
Variables	Pre	Interim	Post	Gain-1	Gain-2	Pre	Interim	Post	Gain-1	Gain-2
Sustained attention	41.13 ± 11.12	59.52 ± 12.21	64.97 ± 10.78	18.39 ± 11.95	5.45 ± 8.59	45.41 ± 16.69	66.34 ± 16.60	65.98 ± 15.23	20.93 ± 13.94	−0.37 ± 10.19
Working memory	51.40 ± 12.33	55.65 ± 10.56	61.19 ± 11.60	4.25 ± 11.23	5.57 ± 7.32	41.83 ± 10.84	61.32 ± 13.37	63.83 ± 12.94	19.49 ± 10.69	2.50 ± 9.44
Adjustment (total)	24.23 ± 6.94	20.19 ± 5.68	19.74 ± 5.25	−4.03 ± 6.92	−0.45 ± 6.16	21.98 ± 9.72	19.07 ± 10.96	20.17 ± 10.16	−2.90 ± 9.09	1.10 ± 7.46
Home adjustment	7.65 ± 3.10	6.77 ± 2.92	6.13 ± 2.85	−0.87 ± 2.45	−0.65 ± 2.72	7.29 ± 3.94	6.76 ± 7.85	6.76 ± 4.34	−0.54 ± 4.19	0.00 ± 3.49
Adjustment with self	13.94 ± 3.79	10.19 ± 3.59	10.77 ± 2.81	−3.74 ± 4.21	0.35 ± 3.76	11.80 ± 4.63	10.12 ± 5.47	10.85 ± 4.90	−1.68 ± 5.25	0.73 ± 4.83
Adjustment with friends	0.29 ± 0.82	0.52 ± 1.46	0.48 ± 0.63	0.23 ± 1.43	−0.03 ± 1.47	0.61 ± 1.11	0.41 ± 0.84	0.68 ± 1.42	−0.20 ± 1.25	0.27 ± 1.55
School adjustment	2.35 ± 1.43	2.48 ± 1.44	2.35 ± 1.36	0.13 ± 1.36	−0.13 ± 1.46	2.27 ± 1.55	1.78 ± 1.41	1.88 ± 1.40	−0.49 ± 1.38	0.10 ± 1.20
Psychological well-being (total)	179.26 ± 19.22	183.68 ± 21.00	186.35 ± 19.69	4.42 ± 13.15	2.68 ± 10.70	180.20 ± 20.04	180.24 ± 20.64	180.95 ± 20.68	4.05 ± 17.14	0.71 ± 13.38
Autonomy	31.74 ± 4.48	33.03 ± 4.93	33.94 ± 4.22	1.29 ± 3.41	0.90 ± 3.74	31.76 ± 4.15	32.54 ± 4.20	32.22 ± 4.13	0.78 ± 3.77	0.32 ± 2.96
Purpose of life	27.39 ± 3.35	28.35 ± 4.01	29.16 ± 3.86	0.97 ± 3.91	0.81 ± 3.55	28.17 ± 5.11	27.93 ± 4.82	28.83 ± 4.53	−0.24 ± 4.77	0.90 ± 3.37
Environmental mastery	27.00 ± 2.77	27.97 ± 3.36	27.84 ± 2.92	0.97 ± 3.47	−0.13 ± 2.88	27.20 ± 2.93	26.76 ± 4.19	26.80 ± 3.26	−0.44 ± 3.75	0.05 ± 2.95
Self-acceptance	33.64 ± 5.26	33.84 ± 5.95	33.87 ± 5.85	0.19 ± 3.76	0.03 ± 3.70	33.20 ± 5.64	32.20 ± 4.74	32.32 ± 5.58	−1.00 ± 4.34	0.12 ± 3.66
Positive relations with others	27.06 ± 4.02	27.35 ± 4.10	28.00 ± 4.05	0.29 ± 2.96	0.65 ± 2.93	28.17 ± 5.11	27.51 ± 3.41	27.54 ± 4.16	0.83 ± 3.52	0.02 ± 3.45
Personal growth	32.42 ± 5.21	33.13 ± 4.92	33.55 ± 4.35	0.71 ± 4.27	0.42 ± 3.36	33.20 ± 4.70	33.32 ± 5.23	33.24 ± 4.36	0.12 ± 4.56	−0.07 ± 4.46

##### 2.6.1.2 Parents

Parents who were present for pre and post-assessment were included in the final analysis. An independent *t*-test was calculated on gain scores (post-test scores minus pre-test scores) to compare parents’ perception of their children’s psychological well-being across EG (*n* = 10) and CG (*n* = 21). Normality of parents’ data was checked using Shapiro–Wilk test. Homogeneity of parents’ data was checked using Levene’s test (Supplementary Table S5). Descriptive statistics of parents’ scores at pre and post-assessment, and their gains are given in Table 5.

**Table 5:** Descriptive statistics of parents’ rating scale EG (*n* = 10) and waitlisted CG (*n* = 21).

	EG Pre-test	CG Pre-test	EG Post-test	CG Post-test	EG Gain	CG Gain
Parents’ rating	*M* (SD)	*M* (SD)	*M* (SD)	*M* (SD)	*M* (SD)	*M* (SD)
Autonomy	10.70 ± 1.16	11.00 ± 1.48	11.70 ± 1.25	11.00 ± 1.48	1.00 ± 1.16	0.00 ± 1.73
Purpose of life	10.80 ± 1.40	11.33 ± 2.40	11.30 ± 1.49	10.71 ± 3.00	0.50 ± 0.97	−0.62 ± 2.16
Environmental mastery	14.30 ± 2.26	15.62 ± 2.80	15.40 ± 1.71	15.38 ± 2.89	1.10 ± 2.08	−0.24 ± 1.73
Self-acceptance	11.10 ± 2.33	11.24 ± 1.95	11.80 ± 1.93	11.81 ± 1.75	0.70 ± 2.63	0.57 ± 2.04
Positive relations with others	12.90 ± 1.52	13.33 ± 1.53	14.50 ± 0.71	13.14 ± 1.74	1.60 ± 1.58	−0.19 ± 1.50
Personal growth	10.70 ± 1.89	11.19 ± 1.78	10.30 ± 2.71	11.10 ± 1.76	−0.40 ± 2.95	−0.10 ± 2.10
Psychological well-being (total)	71.00 ± 6.04	73.71 ± 7.84	75.00 ± 4.93	73.14 ± 9.13	4.00 ± 7.06	−0.57 ± 5.32

EG, experimental group; CG, control group; M, mean; SD, standard deviation.

#### 2.6.2 Qualitative analysis

Audio recordings of FGDs (*n* = 14), teachers’ interviews (*n* = 13), and parents’ interviews (*n* = 5) were paraphrased, translated from Marathi to English, and transcribed by the respective team of facilitator and co-facilitator, or interviewer, as per the questions asked, along with verbatim responses and their general observations. Statements relevant to changes due to ānāpāna practice in these transcripts were identified using MAXQDA Analytics Pro (40). A deductive approach was used for further data analysis. Variables in the study were treated as themes to segregate statements from narratives of students, teachers, and parents. An AI-based language model (41) was used to support aspects of the qualitative analysis process, such as identifying sub-themes in each variable and calculating the count of statements per theme and sub-theme. Final interpretive decisions about themes were taken by researchers. Verbatim quotes from the translated notes of FGDs and interviews were used to illustrate qualitative findings about the effects of ānāpāna practice.

## 3 Results

### 3.1 General trends in statistical results

#### 3.1.1 Students

MANOVA results for Gain-1 and Gain-2 of sustained attention, working memory, psychological well-being, and adjustment are shown in Table 6.

**Table 6:** Multivariate test results (Pillai’s trace).

Variables	Value	*F*	Hypothesis *df*	Error *df*	*P*	Partial Eta Squared
Sustained attention and working memory (Gain-1)	0.332	17.182	2.000	69.000	0.000	0.332
Sustained attention and working memory (Gain-2)	0.086	3.236	2.000	69.000	0.045	0.086
Adjustment (Gain-1)	0.171	3.445	4.000	67.000	0.013	0.171
Adjustment (Gain-2)	0.028	0.487	4.000	67.000	0.745	0.028
Psychological well-being (Gain-1)	0.092	1.093	6.000	65.000	0.376	0.092
Psychological well-being (Gain-2)	0.050	0.566	6.000	65.000	0.756	0.050

df, degrees of freedom; F, F ratio; p, significance. Gain-1: interim test scores minus pre-test scores; Gain-2: post-test scores minus interim test scores.

Key findings of the statistical analysis of students’ data are as follows:

MANOVA results showed that there was significant effect on sustained attention and working memory Gain-1 scores [Pillai’s trace *V* = 0.33, *F* (2, 69) = 17.18, *p* < 0.001, partial Eta squared = 0.33] and on Gain-2 scores [Pillai’s trace *V* = 0.09, *F* (2, 69) = 3.24, *p* < 0.045, partial Eta squared = 0.09] across the two groups.

MANOVA results showed a significant effect on adjustment Gain-1 scores across the two groups [Pillai’s trace *V* = 0.17, *F* (4, 67) = 3.45, *p* = 0.013, partial Eta squared = 0.17], whereas MANOVA for adjustment Gain-2 is not significant [Pillai’s trace *V* = 0.03, *F* (4, 67) = 0.49, *p* = 0.745, partial Eta squared = 0.03].

MANOVA results for psychological well-being Gain-1 [Pillai’s trace *V* = 0.09, *F* (6, 65) = 1.09, *p* = 0.376, partial Eta squared = 0.09] and Gain-2 scores are not significant [Pillai’s trace *V* = 0.05, *F* (6, 65) = 0.566, *p* = 0.756, partial Eta squared = 0.05].

Independent *t* tests of Gain-1 and Gain-2 scores of sustained attention and working memory show that, EG students have shown significant gain on sustained attention scores at post-assessment compared to CG students (*t* = 2.56, *p* = 0.006, Cohen’s *d* = 0.61) (Table 7), whereas a reverse result about working memory of CG students is seen at interim assessment (*t* = −5.86, *p* < 0.001, Cohen’s *d* = −1.39).

**Table 7:** Comparison of sustained attention and working memory.

Variables	EG (*n* = 31)	Waitlisted CG (*n* = 41)	*T*	*P*	95% CI		Cohen’s *d*
	*M* (SD)	*M* (SD)			LL	UL	
Sustained attention (Gain-1)	18.39 ± 11.94	20.93 ± 13.54	−0.83	0.205	−8.65	3.58	−0.20
Sustained attention (Gain-2)	5.45 ± 8.59	−0.37 ± 10.19	2.56	0.006	1.29	10.35	0.61
Working memory (Gain-1)	4.25 ± 11.23	19.49 ± 10.69	−5.86	0.000	−20.42	−10.05	−1.39
Working memory (Gain-2)	5.56 ± 7.31	2.50 ± 9.43	1.50	0.069	−1.01	7.14	0.35

EG, experimental group; CG, control group; M, mean; SD, standard deviation; CI, confidence interval; LL, lower limit; UL, upper limit; Gain-1: interim test scores minus pre-test scores; Gain-2: post-test scores minus interim test scores.

Independent *t-*tests for Gain-1 scores of adjustment areas show that EG students had significant improvement in adjustment to self, compared to CG at interim assessment (*t* = −1.79, *p* = 0.039, Cohen’s *d* = − 0.43), but a reverse result was found on school adjustment, where the CG students improved at this time (Table 8).

**Table 8:** Comparison of dimensions of adjustments for Gain-1 scores.

Dimension	EG (*n* = 31)	Waitlisted CG (*n* = 41)	*t*	*p*	95% CI		Cohen’s *d*
	*M* (SD)	*M* (SD)			LL	UL	
Home adjustment Gain-1	−0.87 ± 2.45	−0.54 ± 4.19	−0.40	0.347	−2.02	1.35	−0.09
Adjustment with self Gain-1	−3.74 ± 4.21	−1.68 ± 5.25	−1.79	0.039	−4.35	0.24	−0.43
Adjustment with friends Gain-1	0.23 ± 1.43	−0.20 ± 1.25	1.33	0.094	−0.21	1.05	0.32
School adjustment Gain-1	0.13 ± 1.36	−0.49 ± 1.38	1.89	0.032	−0.03	1.27	0.45
Total adjustment score Gain-1	−4.03 ± 6.92	−2.90 ± 9.09	−0.58	0.283	−5.04	2.78	−0.14

M, mean; SD, standard deviation; CI, confidence interval; LL, lower limit; UL, upper limit; Gain-1: interim test scores minus pre-test scores.

#### 3.1.2 Parents

Comparison of parents in the EG and CG on their ratings of their children’s psychological well-being is shown in Table 9.

**Table 9:** Comparison of parents’ rating of their children on psychological well-being (differences in post- and pre-scores).

Parents’ rating	EG (*n* = 10)	Waitlisted CG (*n* = 21)	*t*	*P*	95% CI		Cohen’s *d*
	*M* (SD)	*M* (SD)			LL	UL	
PWB dimensions							
Autonomy	1.00 ± 1.15	0.00 ± 1.73	1.65	0.109	−0.24	2.24	0.63
Purpose of life	0.50 ± 0.97	−0.62 ± 2.16	1.56	0.130	−0.35	2.59	0.60
Environmental mastery	1.10 ± 2.08	−0.24 ± 1.73	1.89	0.069	−0.11	2.79	0.72
Self-acceptance	0.70 ± 2.63	0.57 ± 2.04	0.15	0.882	−1.63	1.89	0.06
Positive relations with others	1.60 ± 1.58	−0.19 ± 1.50	3.05	0.005	0.59	2.99	1.17
Personal growth	−0.40 ± 2.95	−0.10 ± 2.09	−0.33	0.743	−2.18	1.58	0.13
Psychological well-being (total)	4.00 ± 7.05	−0.57 ± 5.32	2.01	0.053	−0.07	9.22	0.77

M, mean; SD, standard deviation; CI, confidence interval; LL, lower limit; UL, upper limit; EG, experimental group; CG, control group.

Key findings in parents' data analysis are as follows:

A significant difference was observed for one of the dimensions of psychological well-being, which is positive relations with others, as reported by parents. Parents in the EG reported higher scores than those in the CG (*t* = 3.05, *p* = 0.005, Cohen’s *d* = 1.17).

Table 9 also shows that parents in the EG showed a higher total psychological well-being mean score than those in the CG. Still, the difference was not considered statistically significant, as the 95% confidence interval for the mean difference included zero (95% CI: −0.07 to 9.22), indicating considerable uncertainty in the estimate.

### 3.2 Qualitative findings

#### 3.2.1 General trends

In each of the study variables, there were statements in which the respondents (students, teachers, parents) have suggested that the improvement seen in this variable is due to ānāpāna practice, and there were also statements where respondents have suggested that the improvement is possibly not due to ānāpāna practice. In students’ and teachers’ narrations, total statements about improvement related to ānāpāna were 513 (89%) and statements about improvement not related to ānāpāna were 66 (11%). Statements related to the latter category are further divided into “neutral” or “improvement due to factors other than ānāpāna practice.” Neutral statements are those wherein the respondent does not speak of any improvement. Example of a neutral statement from a student (EG-post-FGD-Group 4), “I was quiet earlier and I am still the same.” Statements related to “improvement due to factors other than ānāpāna” indicate that there is a difference, but show that it is due to factors other than ānāpāna practice.

Factors other than ānāpāna practice mentioned by students are better self-acceptance due to support from parents and sister, and improvement in sports due to practice. Teachers have mentioned that changes in teaching style, familiarity with the teacher, increased seriousness of studies among students being close to 10th grade, strict disciplinary action in case rules were broken, changed sitting arrangement, dialogue with parents, as possibly responsible for improvement among students other than ānāpāna practice. Parents felt that improvement in their children might be due to maturity with age, and support from the teacher, along with ānāpāna.

An observation from all FGD facilitators and co-facilitators was that in post-FGD, EG students were less positive and enthusiastic compared to interim FGDs to share the benefits of ānāpāna practice. Possible reason, as came out in the interaction with students, might be that the FGDs were scheduled by school authorities in place of their sports period. They did not like that they had to miss the sports period for the discussion.

There are some variables and some time-points where neither of the respondents has shared anything. For example, EG students’ narrations do not show anything related to “working memory” at post-FGD. Teachers have not shared anything related to “home adjustment” in either the interim or post-interviews. School adjustment is the clearest example where teachers’ insights are detailed and multi-dimensional, whereas students’ statements are fewer and more individual-centered. As there are very few statements from parents’ narration, emerging trends in their narration were identified, instead of finding sub-themes per variable.

**Figure 2:**
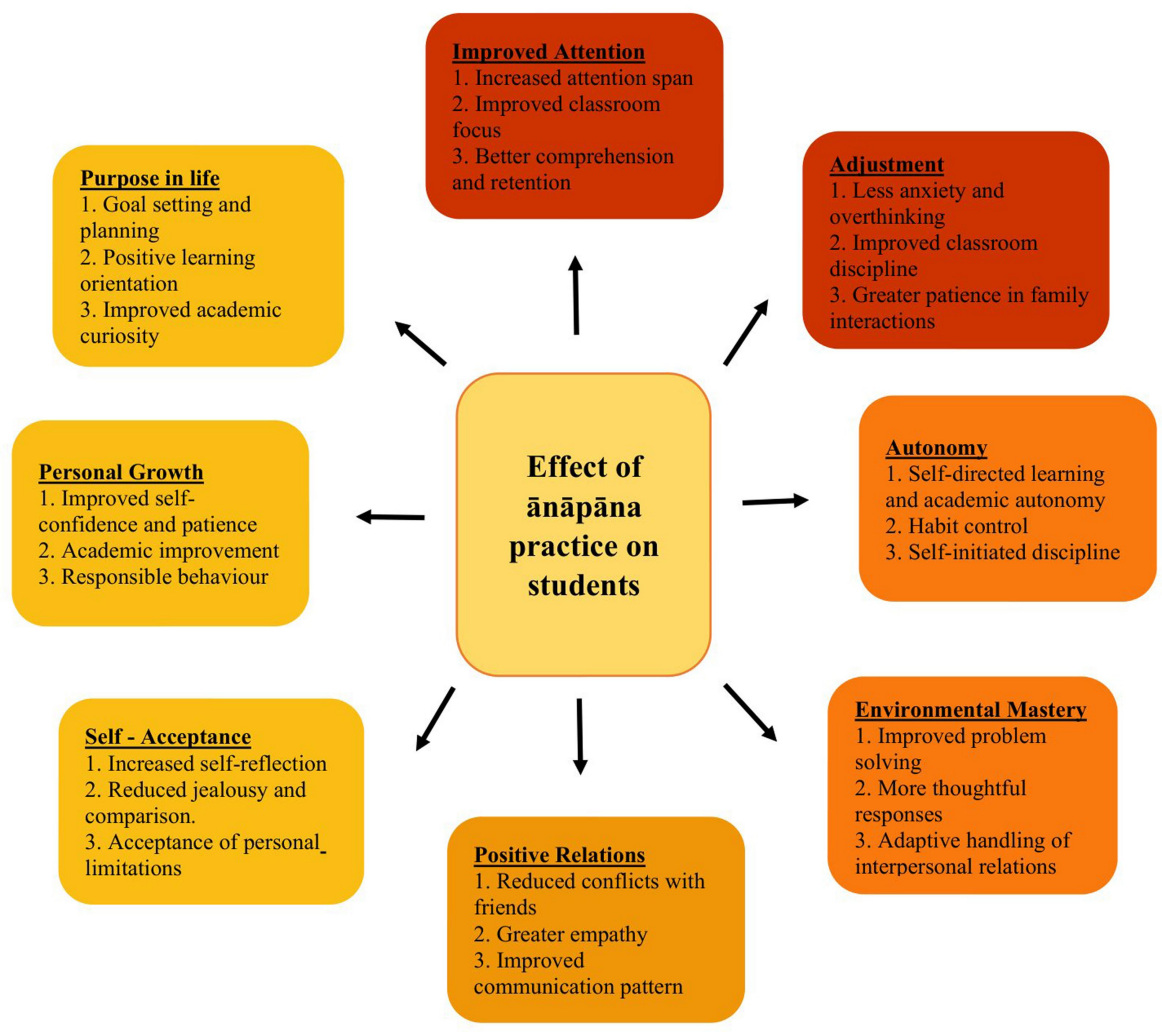
Effect of ānāpāna practice on students: qualitative findings. FGD with students and teacher’s interview.

Key qualitative findings are as follows:

Narratives from students, teachers and parents show the effect of ānāpāna practice on eight main areas of students’ behavior namely improved attention, adjustment, autonomy, environmental mastery, positive relations, self-acceptance, personal growth, and purpose in life. Positive effects in each of these areas are summarized in Figure 2.

**Figure 3:**
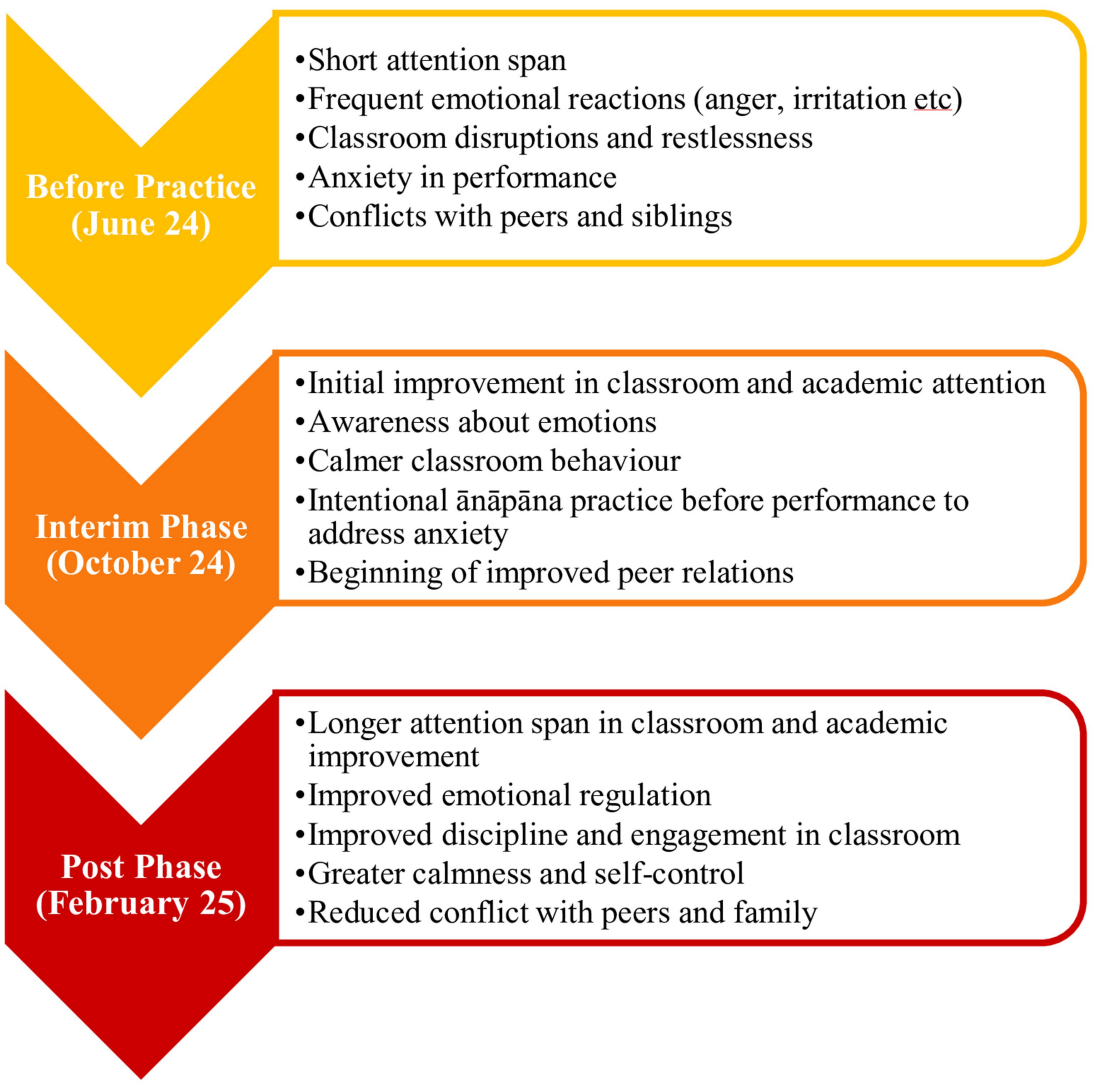
Progression in experimental group students with ānāpāna practice. FGD’s with students from Experimental Group students and interviews of their teachers.

Among the narratives of students and teachers from EG, who practiced ānāpāna over the entire year, a progression of benefits in different areas was observed. Key areas in this trajectory are summarized in Figure 3 and elaborated further in the next section on variable-wise findings.

### 3.3 Variable-wise findings

In this section, findings on each variable, including the independent variable, the “ānāpāna practice,” are elaborated. Findings from quantitative and qualitative data on each variable are integrated to get a better understanding of the effect of ānāpāna on each of the DVs.

#### 3.3.1 Ānāpāna practice

Out of the 89 days between the pre-assessment and interim assessment, ānāpāna practice sessions were conducted for the EG only for 37 days. Of the 90 days between interim assessment and post-assessment, ānāpāna sessions for EG were conducted on 42 days and for CG on 63 days. Ānāpāna was missed on other days either due to school holidays, during online mode of teaching, or when the school schedule was modified for some unavoidable reasons or when there was very low motivation to practice by students.

The number of ānāpāna practice sessions attended by EG students ranged from 37 to 77. The number of ānāpāna sessions attended by CG students ranged from 33 to 61. Details are summarized in Table 10.

**Table 10:** Group-wise number of ānāpāna practice sessions.

Ānāpāna sessions	Experimental group	Control group
Conducted in Semester I	37	00
Conducted in Semester II	42	63
Total	79	63
Range of attended sessions	37–77	33–61

##### 3.3.1.1 Ānāpāna perceived as punishment

The EG students had not appreciated that ānāpāna practice had started only for their class initially. During interim FGDs many students shared that they perceived it as “punishment to improve them” to begin with. One girl from group 4 shared examples of how two different teachers compared their classes to students in other classes. Teachers had told them that, as this class is very mischievous and disobedient, so to improve their concentration, ānāpāna is introduced to them. So, they had started thinking, “why only our class?”.

Class teacher of this class confirmed this perception in her interview during the interim phase: “When children posed the question of “why ānāpāna has been introduced only to us? are we very bad?” I convinced them by saying, “What bad will happen by doing this? Only good thing would happen?” This perception was mentioned as a big challenge to conduct regular ānāpāna sessions by the class teacher. Even in post-FGD, one of the girl participants had mentioned about this. Students’ and teachers’ narrations reveal that not all students liked that they missed assembly due to ānāpāna practice.

##### 3.3.1.2 Disturbance during ānāpāna

Students and the ānāpāna conducting teacher had shared that initially, some would open their eyes or make noise during the practice. However, both the respondents report that the “noise” during ānāpāna got reduced over time. Some students from EG reported that they continued to practice despite distractions as they experienced the benefits of the practice. Students shared that over the time, moments of focus on breath increased and mind wandering reduced, while the teacher observed more stability in their class during the ānāpāna practice. At the interim assessment, one boy from group 3 (EG) says: “boys who used to make lot of noises during ānāpāna earlier, have reduced making noise drastically.”

##### 3.3.1.3 Dealing with disturbing students during ānāpāna practice

In the initial phase, the class-teacher (EG) monitored the disturbing students closely so that they would not disturb others, but there was no need for that later. At the interim interview, she mentioned: “I observe difference in some of the students (names told) - Earlier they would fidget and also disturb their neighbors during ānāpāna but now they sit quietly. In the initial phase I would make them sit separately or I would sit next to them so that they do not disturb others, but I don’t have to do that anymore. Otherwise, there is no difference observed in their behavior in the class.”

The CG class teacher had a different way of dealing with disturbing students. She shared, “(names of students) these boys would laugh and were fidgety and disturb others, I had to scold them initially and threaten them that I would make them leave the class. They gradually understood and their fidgetiness was reduced. Though it is still there, it is not very consistent.”

##### 3.3.1.4 Ānāpāna practice at home

Initially, most of the students were not doing the second time practice to be done at home due to lack of time or motivation, despite reminders by class-teachers. However, as they experienced benefits, they started becoming more regular. The class teacher of EG had shared, “The students who practice at home are mostly sincere students and also because they get support from their parents as well. Their parents are also serious about the practice.” Most of the students from both the classes have confirmed that initially they practiced at home, but later stopped. The CG class teacher shared that, *in certain cases siblings were also encouraged to sit when students practiced at home.*

##### 3.3.1.5 Different attitude toward ānāpāna practice

Two girls from EG admitted during the interim FGD that initially nobody took the practice seriously. But now, everyone has started taking it seriously because of the changes observed (group 5). At the post-interview, the EG class teacher mentioned: “Some of the sincere students are willing to practice and also proactively remind me about it. Some of the students have accepted it as regular activity and comply to the instructions of the teacher. Some were bored and reluctant to practice.”

#### 3.3.2 Attention (sustained attention and working memory)

In qualitative analysis, improved attention emerges as one of the strongest and most consistently reinforced impact areas of ānāpāna practice across groups and time-points, which supports statistical results about this variable (Section 3.1.1). As shown in Figure 2, attention-related benefits reported frequently among students’ and teachers’ narrations are increased attention span, improved classroom focus, and better comprehension and retention. Sub-themes emerged related to sustained attention and working memory, and the frequency of their occurrences in the narrations of students and teachers is given in Supplementary Table S6. Emerged sub-themes show that improved attention has started resulting in academic benefits and emotional regulation.

A pattern is observed among the EG students who were introduced to ānāpāna practice 3 months before the CG students. Through their initial statements, it is evident that attention has improved, which is convincing them to practice ānāpāna. As students’ focus on breath during ānāpāna practice increased, there was better focus on studies as well. It improved their concentration. There was better memorization, reduced forgetting, and better retention. So-called difficult subjects like mathematics and science became easier. Overall, there was better performance in academics, sports, and arts. Success in performance led to more willingness to study. There was more consistency and better study habits. One boy (EG) developed strategic use of ānāpāna to reduce stress before the exam after encouragement from his mother. He shared during the interim FGD: “During exams there is lot to study. In such state we are unable to comprehend what we are studying. My mother advised me to practice ānāpāna and I did ānāpāna for 5 minutes and then I felt better and was able to follow what I was studying. This has not happened once but many times.”

During the interim assessment, teachers teaching the EG shared that students’ attention span has improved compared to last year. Students could now focus longer. The number of inattentive students has reduced. Fewer scoldings and repetitions were needed in the classroom. Students are seen trying to complete work more seriously than before. One teacher has observed that calmness and attentiveness were more visible in morning sessions immediately after ānāpāna practice. She (EG-interim interview-language teacher) shared, “When they meditate in the morning, changes can be seen in the immediate class. There is more calmness then. Students are quiet from within. This calmness reduces as school progresses. If students can meditate even after the lunch-break, it would be better”. In the second semester, improvement in concentration continued, which resulted in better grasping, except for a few weak students who still required repeated explanation.

In the post-assessment, the class teacher of the CG, who had started practicing ānāpāna by then, shared, “This class has good grasping power, everyone including those naughty ones. Possibly because they have good grasping, that is why they are more mischievous. Those naughty students can answer questions the next day, even though they had not paid attention the previous day.” Despite the inherently better attention of CG, EG showing statistically significant improvement in sustained attention by the end of the program strongly indicates the effect of ānāpāna practice on sustained attention. CG students scoring significantly more than EG students on working memory during the interim assessment can be explained by the fact that working memory was the only variable where both the groups were not equivalent on pre-test scores (Section 2.6.1, Supplementary Table S1).

#### 3.3.3 Mental well-being

Mental well-being has been assessed on six dimensions of psychological well-being of Ryff’s model (self-acceptance, purpose in life, autonomy, positive relations with others, environmental mastery, and personal growth) and four dimensions of adjustments (adjustment to self, to school, at home, and with friends). Among these 10 dimensions, only adjustment to self has shown statistically significant improvement among EG students by the end of the first semester, while the CG was still waitlisted. As CG also started practicing ānāpāna, there was no significant difference between the two groups at post-assessment on this dimension. A possible reason might be that CG started showing early changes in adjustment to self because of ānāpāna practice, as revealed through their narrations. Adjustment with self, as assessed by NSCT in this research, is a combination of an adolescent’s attitude toward their own abilities, emotional functioning, coping with emotions, guilt feelings, and goals for the future. No other mental well-being-related variables showed any statistically significant change due to ānāpāna practice. Despite these statistical results, sub-themes related to all six dimensions of psychological well-being and all four dimensions of adjustment have emerged in qualitative findings. Sub-themes in each of the dimensions of psychological well-being as revealed in students’ and teachers’ narratives at different time-points are listed in Supplementary Table S7. Sub-themes related to the four dimensions of adjustment are listed in Supplementary Table S8.

In students’ narrations, sub-themes of psychological well-being cluster around emotional development and positive relations with others. “Emotional development” narrations referred to calmness, relaxation, peace of mind after ānāpāna practice, reduction in unwholesome emotions like anger, restlessness, irritability, impulsivity, stress, nervousness, fear, and boredom; improvement in wholesome emotions like patience, forgiveness, empathy, confidence, resilience, cooperation, greater self-control, and emotional restraint; and reflection, awareness, and acceptance of mistakes. Possibly reflection, awareness and acceptance of mistakes (part of self-acceptance) would have facilitated other aspects of emotional development. How reflection has facilitated other changes in emotional regulation can be seen in the statements segregated into the two dimensions related to self-process, “self-acceptance” and “adjustment to self.” Both these dimensions show a clear developmental trajectory among EG students from the interim to the post-assessment.

##### 3.3.3.1 Self-acceptance

The main benefits observed in self-acceptance are increased self-reflection, reduced jealousy and comparison, and acceptance of personal limitations (Figure 2). Self-Acceptance is more richly articulated in students’ self-reports than in teachers’ accounts. Students emphasize internal psychological shifts, recognizing emotions, accepting limitations, and letting go of defensiveness, while teachers identify outward behavioral markers such as accountability and acceptance of feedback.

During the interim FGD, students’ reflections primarily center on emerging self-awareness and introspection. One girl student (EG-interim FGD-Group 4) articulated this shift by sharing, “We generally look at others and what they like, but during ānāpāna I explored what is my foundation… When I have a fight with my mother or a friend, I get a solution in ānāpāna. It helps me reflect and realize where I have gone wrong or if I have had ego.” This account illustrates an early movement toward inward focus, recognition of personal emotions, and the beginnings of non-defensive self-evaluation, though without yet indicating stable emotional acceptance. By the post-assessment, students’ narratives increasingly reflect consolidated self-acceptance, characterized by greater emotional ownership, reduced irritability, and acceptance of differing perspectives. Statements such as “I have accepted that we both have different perspectives” (EG-post-FGD-Group 5) suggest a transition from recognizing internal reactions to accommodating them with clarity and restraint. This progression indicates a shift from awareness of self toward acceptance of self. In the CG, students’ accounts point to emerging self-acceptance, particularly in letting go of rejection or possessiveness.

##### 3.3.3.2 Adjustment to self

Qualitative findings about adjustment to self also cluster around emotional regulation and overlap with findings related to self-acceptance. In the interim assessment, it is found that there is general calmness, an initial reduction in irritation, and early efforts to control emotions. This is seen to evolve into more specific, intentional self-regulation (anger pause, meditation during conflict, and managing thoughts) in the post-phase. The students, based on their own experience, have developed their strategies as expressed by one of the students in the post-phase (EG-post-FGD-Group 5), “I meditate sometime when I am irritated or have had arguments. I meditate so that I can calm down.” Students shifted from emotional reactions to deliberate emotional management. CG students have reported improvements, but their statements are less rich in detail, less clearly connected to mechanisms and more generalized (“mind more stable,” “anger reduced”).

##### 3.3.3.3 Positive relations with others

Emotional development has resulted in positive relations with others, shown in openness in relationships, thinking before reacting, calmer communication, reduced conflict, thoughtful conflict resolution, letting go of grudges, attempts to forgive, reciprocal sharing with siblings, social maturity by trying to understand the perspective of others, and positive relations at home and with friends. A sharing of a girl student (EG-post-FGD-Group 3) “When I have an argument with a friend, I choose to forgive and wait until they have calmed down before communicating. I give them time before having a conversation” indicates improved communication patterns. These relational improvements emerged in varied settings, including peer interactions, family relationships, and cooperative exchanges in academics and sports. One of the students (EG-post-FGD-Group 2) mentioned his experience while playing football, “While playing I have improved ability to understand other person’s point of view. Earlier I would get angry if someone did a formation in football which is not as I want. Now I try to understand. Earlier I would shout. Now I don’t. I am more co-operative now. I am also able to perform better in online games as well.” These changes are also reflected in other study variables, which are adjustment at home and adjustment with friends.

##### 3.3.3.4 Autonomy and environmental mastery

Better emotional regulation has influenced other dimensions of psychological well-being like autonomy and environmental mastery. As shown in Figure 2, benefits observed in autonomy are self-directed learning, academic autonomy, habit control, and self-initiated discipline. For example, one boy student (EG-post-FGD-Group 1) said: “Earlier my parents would take my studies but now I can do it myself.” Teachers observed that “students independently asked doubts, discussed academic topics, and engaged in self-study,” indicative of academic autonomy. Autonomy is seen as developmental by parents across both groups, with clear ānāpāna attribution appearing only in one CG parent’s interview.

Benefits in environmental mastery are observed in improved problem-solving, more thoughtful responses, and adaptive handling of inter-personal relations (Figure 2). One girl student (EG-interim FGD-Group 5) shared how emotional regulation has resulted in better handling of situations. A change depicting improvement in emotional regulation is seen in her narration, “Earlier I used to feel panic while handling certain situations but now it has improved. I take decisions with a calm mind.”

##### 3.3.3.5 Personal growth and purpose in life

*Personal growth* dimension is revealed in academic improvement, responsible behavior, and emotional development (Figure 2). A clear progression is visible among the EG students. In the interim FGD, EG students reported foundational changes such as increased patience, study endurance, and emerging confidence. In the post-FGD, these early shifts expanded into more differentiated forms of personal growth, including emotional regulation, creative expression, persistence, and structured academic engagement. One of the students (EG-interim FGD-Group 4) shared her experience about persistence, “I do sketching and painting, and earlier I used to give up after making mistake in the drawing, now I try to correct and improve it.” Students from CG, at post-FGD, reported improvements primarily in outcomes (marks, sports performance, maturity), suggesting early-stage developmental shifts aligned with shorter exposure to ānāpāna practice.

The dimension, “*purpose in life*,” is seen less clearly in students’ narrations; at the most, they are expressed in a goal-oriented approach to study. Purpose-related reflections surfaced later than other attributes, becoming more visible by post-assessment among EG students. Teachers confirm more seriousness, curiosity, and responsibility among many students of EG by the second semester.

##### 3.3.3.6 Mental well-being: teachers’ observations

Teachers of EG have shared how students were more settled and that the classroom became calmer by the end of the second semester. Naughty students were still present, but overall, the class was more manageable. Reduced anger was observed among few specific students. The class teacher of EG reported in the interim phase that, “As the class is noisy, I have to constantly instruct them to ‘keep quiet’ or ‘save energy.’ In last 3 months, I still gave these instructions (10–15 times) but the frequency/number of times I had to give this instruction has reduced (4–5 times) than before.” In the post-assessment, the teachers have provided varied and detailed observations pointing out continued settling of the class, fewer fights, and calmer behavior, but also persistent challenges among a smaller subgroup of students. The class teacher (EG), during the post-interview, quoted the experience of two other teachers about this class, “There were days when they did not have to scold the children and the teachers were happy about it. And now they are able to teach more peacefully. One of them has observed that the class seems to be more settled than it was in the first term.” Similar feedback was received in the interview of the language teacher who taught both the classes. She shared, “*not CG, but EG students have definitely become calmer. I think, it requires time for effects of meditation to show*.” Teachers of CG have shared that the classroom atmosphere in the second semester became calmer and there was reduced tension in class. Physical fights had reduced, but verbal aggression and abusive language were still present. The majority of the students have shown sincerity and seriousness toward study and readiness to learn and do activities, though a small group of students still lack clarity. Teachers from both the classes have shared that there was more acceptance of mistakes by students.

##### 3.3.3.7 Adjustment

Main qualitative findings about adjustment are improved classroom discipline, greater patience in family interactions, and reduced anxiety and overthinking (Figure 2). Findings about adjustment to self are already elaborated above. Adjustment to school, adjustment at home and adjustment with friends overlap with qualitative findings related to different dimensions of psychological well-being and have been mentioned at appropriate places above.

## 4 Discussion

The present research project was designed and carried out with the main objective to evaluate the psychological effects of ānāpāna practice, the first step of vipassanā meditation, introduced in the framework of the MITRA program for school-going students in 8th grade (a later phase of early adolescence). MITRA program for school students, a joint initiative of Maharashtra Government and the VRI since 2011, is considered as having a salutogenic orientation, as it fulfills the three criteria of such orientation by Antonovsky (17, chap. 29).

1. Through the MITRA program, ānāpāna practice is introduced for all school-going students of 5th to 10th grades, irrespective of their gender, socioeconomic background, level of intelligence, and disabilities or behavioral issues.
2. The MITRA program is not for reducing any psychosocial risks like bullying, but is for developing a salutary factor, namely mastery over the mind.
3. Though ānāpāna practice leads to enhanced concentration and mindfulness, it is not a retraining method specifically for these abilities. Instead, it is supposed to provide a skill for resisting temptations and developing healthy habits, both of which facilitate happiness and satisfaction in a person’s life.

Regular ānāpāna practice is supposed to develop a generalized resistance resource (GRR) for school-going adolescents. The term GRR, coined by Antonovsky, refers to the resources of a person, group or community that facilitate an individual’s ability to cope effectively with stressors (17, chap. 12). The importance of mastery over mind by training attention has been acknowledged widely as an important resource for a child. A pioneering figure in psychology, William James (1842–1910) wrote in his famous book, “Principles of Psychology” (42, p. 957), “The faculty of voluntarily bringing back a wandering attention over and over again is a root of judgment, character and will. An education which should improve this faculty would be education par excellence.” However, he has not mentioned any specific method to improve this faculty of attention. “Voluntarily bringing back a wandering attention over and over again” is the exact process of ānāpāna meditation. In fact, through the teaching of ānāpāna practice in the MITRA program, young school-going children are introduced to an important cultural resource for dealing with omnipresent stress in life and for living a happy life, which is vipassanā meditation, the systematic teaching of the noble path for the eradication of suffering as taught by the Buddha.

It is well-known how since the seminal MBSR, the potential of Buddha’s teachings for the eradication of suffering is being utilized in the field of mental health (10, 11). However, adoption and adaptation of vipassanā meditation for therapeutic interventions might lead to a general impression that vipassanā is for people suffering from some “problems” and not for everyone. In recent years, especially since the beginning of positive psychology (43), mindfulness’s potential for going beyond stress reduction and for fostering well-being is also being acknowledged. Still, in a systematic review of mindfulness-based positive psychology interventions by Allen et al. (44), it was found that nature, pattern, duration, and focal area of these interventions varied greatly and mostly centered around a few specific positive variables rather than overall well-being and flourishing. The MITRA program, wherein ānāpāna is taught as a technique to “gain mastery over mind” for a happy life in an educational set-up, may prove to be an antidote to such reification of vipassanā.

Findings from this study using mixed methods have led to important insights regarding ānāpāna practice for adolescent students and for the MITRA program. According to the experiences of MITRA volunteers in the last decade, the effects of regular ānāpāna practice among school students can be seen within 2–3 months (Chandrashekhar Datye, MITRA coordinator, Pune, personal communication, June 2024). As anticipated by MITRA volunteers, the psychological effect of ānāpāna practice was observed within the first 3 months (30+ sessions of ānāpāna) on adjustment to self among EG students compared to the waitlisted CG in the present study. EG students’ narrations in interim FGDs and their teachers’ observations are indicative of early signs of emotional development. It can be seen in sub-themes like awareness of emotions, calmer classroom behavior, intentional practice of ānāpāna before performance to reduce anxiety, and the beginning of improved peer relations (Figure 3). At post-assessment, sub-themes like improved emotional regulation, improved discipline and engagement in classroom, greater calmness and self-control, and reduced conflicts with peers and family in the narrations of these students and teachers show that the progression continued. However, at post-assessment, gains in scores of adjustment to self among EG students were not statistically significant as compared to CG students who had started practicing ānāpāna by then. Both the results together suggest that improvement in some aspects of mental well-being is an early sign of the psychological effects of āṇāpāna practice by young students in early adolescence.

Based on the review of empirical and theoretical literature related to the process, effect, and mechanism of ānāpāna and vipassanā meditation, researchers had a hunch that attention-related variables (sustained attention and working memory) would be the first to show the effect of ānāpāna practice among the DVs (attention and mental well-being) in the present study. Themes such as increased attention span, improved classroom focus, better comprehension, and retention did come up in the narrations of students and teachers of EG at interim FGDs (Figures 2, 3). However, psychometrically noticeable changes in sustained attention, as assessed by the FCT, could be seen only after 6 months (70+ sessions of ānāpāna) (Table 7). As the two groups were not equivalent on working memory pre-test scores on the digit symbol substitution test (DSST) (Supplementary Table S1), it is understandable that the CG have gained significantly more on working memory at the interim assessment.

In a latest study by Londhe et al. (45), the effect of ānāpāna practice as taught by SN Goenka on the sustained attention of students in 6th and 7th grade was assessed using a research design similar to the present study in a school in Bangalore, India. Students in EG practiced ānāpāna one time every day during school assembly, whereas CG students attended a separate assembly. There used to be an interactive session with EG students at regular intervals to encourage them for ānāpāna practice and clear their doubts, if any. These sessions were conducted by a person who was a regular vipassanā practitioner. Participants’ sustained attention was measured using the number cancelation test (NCT) at baseline, after 3 months, and after 6 months. Scores on the NCT were based on total cancelations, correct responses, omissions, errors, and qualitative response patterns (systematic or haphazard). Results showed significant improvement in EG students with reduced omissions and errors, and a moderate effect size of 0.39 even after 3 months’ ānāpāna practice. This trend of improvement continued in the post-assessment after 6 months. Comparison of the present study with this study shows that regular interaction with participants to discuss their ānāpāna-related doubts might be an important intervening variable between ānāpāna practice and its effect on attention.

As mentioned in the Introduction, many studies have reported the usefulness of breath awareness, which is a part of mindfulness-based interventions, for improving mental well-being (12, 30). In a recent study, Pradhan and Bhutia (46) have investigated the effect of the practice of ānāpāna on the well-being of secondary school students in Kalimpong, India, using a mixed-methods design. They found improvement on different dimensions of well-being (emotional, psychological, social, spiritual, and self-awareness) as assessed by the well-being index of Chouhan and Sharma (47) after 21 days of ānāpāna practice. The ānāpāna practice instructions were prepared by a happiness and well-being coach with 8 years of experience in the field. The ānāpāna practice in this study had three phases: (1) being aware of breath by focusing on the tip of the nose, (2) observing breath through box-hand breathing or counting breath, and (3). watching breath using techniques taught in the first two phases. Thus, though this article reports the effect of ānāpāna practice on the well-being of adolescents, the ānāpāna instructions are a combination of grounding, breath anchoring, and mindfulness practice. The present study is unique in assessing the effect of only “breath awareness,” without combining it with any other mindfulness techniques. The importance of awareness of natural breath without any verbalization for the purification of the mind in the original Buddhist context has been explained in the Introduction section.

Taking data from multiple sources has proven an advantage in quantitative as well as qualitative aspects of the present study. Despite showing improvement in adjustment to self, students did not show any significant improvement in psychological well-being scores, another measure of mental well-being, in either of the semesters. However, in EG, parents’ rating of their children’s positive relations with others, one dimension of psychological well-being was significantly better at the post-assessment than their counterparts in the CG. In qualitative data, teachers’ reflections about students’ school adjustment were more nuanced than those of the students themselves. In home adjustment, students have shared many things about their relations with siblings and parents, which teachers were not aware of at all. Overall, the quantitative findings in the present mixed-method evaluation have shown that regular ānāpāna practice does have a positive effect on sustained attention and adjustment to self. Themes emerged in the qualitative data from students, teachers, and parents cluster around a better focus on studies, emotional regulation, positive relations with others, and the strategic use of ānāpāna to deal with stress.

Nonetheless, qualitative findings also show possible hurdles to the regularity of ānāpāna practice among adolescents. Table 10 shows that ānāpāna sessions were conducted for EG less than 50% of the days in the academic year, whereas the sessions for the CG neared to 70% of school days. Reasons listed by teachers were school holidays, online mode of teaching, a modified school schedule, and very low motivation to practice by students (Section 3.3.1). Low motivation by students can be explained further through qualitative data. Participants in the present study have shared honestly that practice of observing natural breath was counterintuitive for them. For example, one girl student from EG shared during the interim FGD: “Initially I used to feel, we are so young. This is our time to play and have fun. Why are they making us meditate?” Fortunately, within 2 weeks, students with “ānāpāna not for our age” conception started seeing a difference due to practice and decided to do it willingly. Still, having to miss assembly had led to a negative opinion about this activity among many of them till the post-FGD. Another hurdle was created when ānāpāna was perceived as “punishment” by EG students. This perception was generated due to what some of the teachers told them, possibly to handle the undisciplined classroom behavior. Despite the salutogenic orientation of the MITRA program, in this school it was decided to introduce ānāpāna practice only for two 8th classes, one after another, for research purposes, though school authorities intended to introduce it to the entire school after getting the results of this research. EG students’ wrong perception of ānāpāna practice shows the importance of introducing a program for all students, and shows how stigmatizing process may start if a program is introduced to a few selected students.

In a review by Ergas and Hadar (48) of 447 articles on academic discourse related to mindfulness between 2002 and 2017, two trends in adopting mindfulness in an educational set-up are visible: mindfulness in education, and mindfulness as education. Mindfulness in education is implemented through mindfulness-based interventions, often outsourced, and secularized, that focus on improving mental health, social–emotional learning, and academic performance. It is predominantly effects-oriented and applied across all age groups. Mindfulness as education integrates mindfulness into teaching and learning processes, emphasizing self-knowledge, transformative learning, and critical pedagogy. The MITRA program is similar to the former trend, in which persons outside the school teach ānāpāna meditation to students and teachers. However, after the initial training, regular ānāpāna sessions are conducted by school teachers in this program, which also showed the beginning of the latter trend. It also means that whether the MITRA program remains an externally forced activity or part of the education process depends on the individual motivation of the ānāpāna conducting school teacher. Qualitative findings in the present study have shown that the dynamics between students and teachers became an important intervening variable. Due to student-teacher dynamics, the intervention to develop a GRR, itself triggered stress to some extent for the student participants in the present study.

These findings suggest a necessity for more thorough training for school teachers conducting ānāpāna sessions. In the initial days of the MITRA program, it was a necessary condition that the school teacher who conducts daily ānāpāna sessions should have done at least one 10-day vipassanā course (1). Considering the workload of school teachers these days, attending 70 min of MITRA training is considered sufficient for teachers to conduct ānāpāna sessions for school children (2). A teacher who has attended at least one vipassanā course and is practicing regularly would be in a better position to explain the importance of ānāpāna to students, or to handle their doubts and queries regarding practice. The importance of an established, personal mindfulness practice for the effective teaching of mindfulness-based intervention has been emphasized frequently, even by Jon Kabat-Zinn, the pioneer of mindfulness-based therapeutic interventions in the field of psychotherapy (49).

Perception of ānāpāna practice varied among the student participants. There were a few students who had realized the potential of ānāpāna practice for dealing with anxiety, fear, anger, or a difficult task (EG-interim FGD-group 1 and 3). There were also a few students for whom it was a forced activity and a waste of time. At the interim FGD, students in Group 3 shared that *even now if some submission was there, some students will sit at back of classroom and complete their submission during ānāpāna time.* A similar thing came out in the FGD of this group even at post-assessment. Some boys still seemed to perceive the 10 min spent on ānāpāna as time wasted. One boy shared that *had they done studies during this time then they would have benefited more*. As mentioned by the EG class-teacher (Section 3.3.1.5: different attitude toward ānāpāna practice), some students were proactive, others were just complying with instructions, and a few were against the practice. Different attitudes and benefits are observed even while adults attend vipassanā courses (50), and so is the case with the MITRA program. Better not expect that all the students would readily accept this activity and start practicing regularly immediately. The objective is to introduce them to this technique. How it would be perceived and whether or not it would be practiced regularly depend on many other factors beyond measurement at the moment. Teachers had recorded the number of ānāpāna sessions attended by each student. However, whether the student has focused on breath or not during these 10 min cannot be recorded. The only criterion to know that students are practicing seriously would be if students report the use of ānāpāna intentionally during stressful moments. Fortunately, such use can be seen among narrations of at least some of the students (Section 3.3.2).

Inward focus, reflection, and self-awareness came out as the main processes facilitated by ānāpāna practice in the present findings. In the cognitive training approach to improve attention, a person is supposed to do a training task repeatedly (29). Compared to that, ānāpāna, as a technique that improves attention, is unique because of the inward focus generated, initially by closing the eyes. However, a person may get engaged in various thoughts even with closed eyes. Ānāpāna strengthens inward focus and attention by focusing on the breath and not engaging with thoughts that arise while observing breath. This is very nicely mentioned by one young participant from EG (Section 3.3.3.1: self-acceptance) in this study. Although students had different attitudes to ānāpāna practice, their narrations do reveal self-related processes, such as an inward focus and self-reflection that started due to the practice. Students’ reflections were related to different things happening in their environment, including ānāpāna practice.

Participants in the present study (average age 13 years) were at the end of early adolescence. The major developmental task to be accomplished in this period is to move toward independence from dependence on family (51). The issue addressed in this study was whether and how ānāpāna contributes toward this developmental task of autonomy during adolescence, and whether it helps to mitigate the developmental challenges in any way. Larger and longitudinal data of adolescents practicing ānāpāna would be necessary to speak confidently anything about this aspect. Still, qualitative findings in this study suggest that ānāpāna practice may support the development of autonomy in adolescents by enhancing self-directed learning, academic autonomy, habit control, and self-initiated discipline (Figure 2).

In a meta-analysis, Mettler et al. (52) investigated the strength of mindfulness-based programs’ (MBP) effects on school adjustment and explored the characteristics of effective mindfulness programs. Based on the results, they have reported that MBP are more effective in the late adolescence than in early and middle adolescence. During late adolescence, there was a significant effect of MBP at post-intervention as well as follow-up period, whereas in middle adolescence MBP is found to have a significant effect only at post-intervention. It would be worth exploring how much of the present positive findings are retained at follow-ups.

Ānāpāna practice provides a unique way to deal with stress, which is omnipresent in human life, and a few students had shared having realized and utilizing this potential of ānāpāna. Stressors in students’ lives can be facing exams, different opinions by peers, judgments/scoldings from teachers and parents. When one is stressed about something, the focus of the mind is on the stressor, though without calmness, and hence there is a rush of thoughts leading to confusion. Because of lack of clarity, one is stuck with the stressful aspect of the situation and not able to think of different possible options. Awareness of breath during stressful moments, which activates physiological responses related to relaxation (53), may give flexibility in dealing with stressors. Regular ānāpāna practice strengthens the consistency of breath awareness, and during stressful moments, awareness of breath starts happening automatically. Thus, well-practiced ānāpāna can be a potential coping resource while facing a stressor. Practicing ānāpāna in the face of stress is not running away from the stressor, but is getting prepared to deal with it in better ways. In the MITRA audio tracks (9), ānāpāna is introduced with the metaphor of using a tire-tube while swimming. “As a tire-tube helps while swimming in a swimming pool, awareness of breath helps for living life full of vicissitudes.”

In his salutogenic model, Antonovsky has suggested that persons with a strong versus a weak SOC appraise and react to stressors differently (17, p. 70). SOC, with its three components comprehensibility (understanding), manageability (having resources), and meaningfulness (finding purpose), is a key concept in this model. Lindström and Eriksson (17, p. 118) defined the development of SOC as a lifelong learning process. Strength of SOC was found to be shaped by three kinds of experiences—consistency, underload-overload balance and participation in socially valued decision-making. What kind of experiences one comes across may not be in his/her control, but working on one’s emotions, including reducing negative emotions, developing positive emotions, and developing emotional restraint, is very much within their control. As discussed in Section 3.3.3, reflection, awareness, and acceptance of mistakes facilitated through ānāpāna practice can contribute to emotional development. Based on this evidence, ānāpāna practice might be a possible tool for developing SOC, though thorough research is required in this direction. In a further study, SOC can be taken as a dependent variable to evaluate the effect of ānāpāna or even vipassanā meditation.

As explained in the Introduction section, the key to the salutogenic process in vipassanā meditation is equanimity of mind developed based on direct experience of the impermanence of bodily sensations. A study using interpretive phenomenological analysis of 12 long-term vipassanā meditators (with 4,000+ h of meditation experience) has shown how vipassanā meditation can lead to deeper existential insights (53). Compared to the depth and length of vipassanā meditation learnt and practiced in a 10-day vipassanā course, the findings in the present study (improved focus, calmness of mind, emotional regulation, pause before reacting: Sections 3.3.2 and 3.3.3) facilitated by the MITRA program are just a glimpse of the full potential of vipassanā meditation. Nonetheless, ānāpāna practice, the first of step of vipassanā meditation, is important as an introduction of the valuable heritage of humanity, to adolescents. One never knows: someone from them who are introduced to ānāpāna practice through the MITRA program during their school days may trace the roots of this ancient Indian technique as they face the vicissitudes of life as an adult, attend a vipassanā course and explore the full potential of this ancient mind-cultivating technique.

## 5 Conclusion

Based on the statistical results and qualitative findings in the present study, it is concluded that the MITRA program, in which ānāpāna meditation, a beginning step of vipassanā meditation, is taught to school students, has been a valuable addition to the educational process because ānāpāna practice improved attention, which is foundational for academic and co-curricular activities. ānāpāna practice facilitated inward focus and self-reflection, which led to better self-acceptance and better adjustment with self and others. Ānāpāna helped to build a coping resource with stress for those students who practiced it regularly.

Positive statistical results and qualitative findings in the present study, despite irregular practice and varied attitudes toward ānāpāna practice, indicate greater potential for the MITRA program to improve attention and mental well-being if implemented completely as per the given directions. The present research has shown that initially, students may perceive it as another forced activity in a school setup. However, as they see its benefits, they become more regular in their practice. For better utilization of the salutogenic potential of the MITRA program, more thorough training for teachers conducting ānāpāna practice is considered necessary.

## 6 Limitations and future directions

Though authors were aware that the MITRA program is related to salutogenesis and not pathogenesis while designing this research project; they came to know the details of the salutogenetic model by Aaron Antonovsky only in the writing phase. Hence, the SOC, the key component of the salutogenetic model, was not much pondered upon while designing this research. The use of SOC as a dependent variable in a future study will be useful for further understanding of the role of ānāpāna practice in developing SOC.

A few important suggestions for further research have come forth from the present study. One of them is to explore the influence of “regular interaction with participant students to discuss and clarify doubts related to their ānāpāna practice” while assessing the effect of ānāpāna practice. This can also be useful for ensuring regular practice and for better implementation of the MITRA program. The MITRA program is designed for secondary and higher secondary students. In the present research, we have studied the effects of MITRA for 8th-grade students. Studying the effect of MITRA for other age group students and their comparison would be useful for further spread of the MITRA program. The qualitative data in the present research suggest that not everyone was equally benefited. What factors influence these differential benefits would be worth exploring in further research.

Though this study is one of the initial empirical research projects on the MITRA program, a few limitations must be acknowledged. In view of the number of years since the inception of the MITRA program, the sample size in the present study is small. The entire class was treated as the experimental or the control group, and students were not assigned randomly. The number of ānāpāna practice sessions attended by each participant was varied in the present study. The number of minimal sessions necessary for the effect of ānāpāna needs to be investigated in a further study.

## Data Availability

The raw data supporting the conclusions of this article will be made available by the authors, without undue reservation.

## Footnotes

1 Given in parentheses are Pali words from Buddha’s teachings.

2 Right to Education (RTE) admissions are done by the Government in private unaided schools. Children from economically weaker sections, disadvantaged groups, or children with disabilities (40%+) can apply for 25% RTE admissions. Applicants must be 6–14 years old (or specific pre-primary ages) and residents within 1–3 km of the school.
