# Supplementary material for: Effect of ānāpāna meditation on attention and mental well-being in secondary school students: a mixed-methods evaluation
Source: Front Public Health. 2026 Apr 24;14:1772248. doi: 10.3389/fpubh.2026.1772248 (PMC13154396; doi:10.3389/fpubh.2026.1772248)
Supplement: Supplementary file 1 [file Data_Sheet_1.pdf]

Supplementary Table 1

Equivalence of Experimental and Control groups on pre-test scores

Variables	**EG (*n*=31)**	**Waitlisted CG (*n*=41)**	***t***	***P***	**95% CI**	
	M SD	***M SD***			**LL**	**UL**
Sustained attention	41.13 ± 11.12	45.41 ± 16.70	-1.24	0.221	-11.20	2.63
Working memory	51.39 ± 12.33	41.83 ±10.84	3.49	0.001	4.10	15.03
Adjustment	24.23 ± 6.94	21.98 ± 9.72	1.09	0.277	-1.85	6.35
Psychological wellbeing	179.26 ±19.22	180.20 ± 20.04	-0.20	0.842	-10.29	8.41

EG = experimental group, CG = control group, *M* = mean, *SD* = standard deviation, *CI* = confidence interval, *LL* = lower limit, *UL* = upper limit

Supplementary Table 2
Normality assessment of study variables using Shapiro–Wilk test
(EG: n = 31) & (CG: n = 41)

**Variables**	Sustained attention		Working Memory		Psychological Wellbeing		Adjustment	
**Group**	EG	CG	EG	CG	EG	CG	EG	CG
**Statistic**	0.954	0.965	0.977	0.967	0.979	0.961	0.978	0.900
p	0.206	0.226	0.740	0.284	0.788	0.170	0.744	0.002

EG = experimental group, CG = control group

Supplementary Table 3: Tests Homogeneity of Variances

Box’s M test of homogeneity of covariance

**Test**	**Box's M**	***F***	**df1**	**df2**	***P***
Sustained attention and memory (Gain-1)	1.107	0.357	3	535396.7	0.784
Sustained attention and memory (Gain-2)	4.260	1.375	3	535396.7	0.248

df = degrees of freedom

Supplementary Table 3.1
Levene's test of homogeneity of variances for all study variables
(Gain-1 = Interim test scores minus pre-test scores)

**Variables**	Levene Statistic	*df*1	*df*2	*p*
Sustained attention	1.727	1	70	0.193
Working Memory	0.025	1	70	0.874
**Adjustment**				
Home Adjustment	3.465	1	70	0.067
Adjustment with Self	3.188	1	70	0.078
Adjustment with Friends	0.012	1	70	0.913
School Adjustment	0.412	1	70	0.523
Total Adjustment score	1.810	1	70	0.183
**Psychological Wellbeing**				
Self-acceptance	0.485	1	70	0.488
Autonomy	0.000	1	70	0.994
Positive relations with others	0.220	1	70	0.641
Environmental mastery	0.121	1	70	0.729
Personal growth	0.315	1	70	0.576
Purpose of life	0.590	1	70	0.445
Psychological wellbeing (total)	0.336	1	70	0.564

df = degrees of freedom, *p:* significance

Supplementary Table 3.2
Levene's test for homogeneity of variances for all study variables
(Gain-2 = post test scores minus interim test scores)

**Variables**	**Levene Statistic**	***df*1**	***df*2**	***P***
Sustained attention	0.181	1	70	0.672
Working memory	1.140	1	70	0.289
**Adjustment**				
Home Adjustment	1.712	1	70	0.195
Adjustment with self	2.951	1	70	0.090
Adjustment with friends	0.521	1	70	0.473
School adjustment	2.378	1	70	0.128
Total Adjustment score	1.396	1	70	0.241
**Psychological wellbeing**				
Self-acceptance	0.004	1	70	0.952
Autonomy	0.865	1	70	0.355
Positive relations with others	0.039	1	70	0.844
Environmental mastery	0.328	1	70	0.569

Personal growth	1.290	1	70	0.260
Purpose of life	0.343	1	70	0.560
Psychological wellbeing (total)	1.217	1	70	0.274

df = degrees of freedom, *p* = significance

Supplementary Table 4
Pearson correlations between main study variables

Variables	Sustained attention	Working memory	Psychological wellbeing	Adjustment
Sustained attention	1			
Working memory	.199	1		
Psychological wellbeing	.182	.108	1	
Adjustment	-.231*	.016	-.212	1

*$P < .01$.

Supplementary Table 5
Normality and homogeneity of variance of Parents' data

	**Shapiro-Wilk test**					
**Variables**	**Experimental group (n=10)**		**Control Group (n=21)**		**Levene's test**	
	Statistic	*p*	Statistic	*P*	F	*p*
Autonomy	0.953	0.703	0.947	0.301	1.129	0.297
Purpose of Life	0.868	0.095	0.926	0.112	5.333	0.028
Environmental mastery	0.892	0.180	0.950	0.343	0.675	0.418
Self-acceptance	0.908	0.269	0.921	0.091	3.104	0.089
Positive relations with others	0.838	0.042	0.895	0.029	0.538	0.469
Personal growth	0.927	0.419	0.935	0.174	1.677	0.206

Supplementary table 6:<br>Themes and sub-themes related to sustained attention and working memory				
**Assessment phase**	**Respondents**	**Themes**	**Subthemes**	**Frequencies**
Interim	Students	Sustained attention	Academic benefit due to improved concentration<br>Improved focus and reduced distractions<br>Improvement in cocurricular activities<br>Emotional regulation leading to better concentration<br>Behavior-linked indicators of attention<br>Mental stillness and sustained attention<br>Neutral	41
Interim	Teachers		Improved attention span<br>Behavioral regulation supporting attention<br>Improved grasping	07
Post	Students		Academic benefit due to improved concentration (EG & CG)<br>Improved focus and reduced distractions (EG & CG)<br>Improvement in cocurricular activities (EG& CG)<br>Emotional regulation leading to better concentration (EG & CG)<br>Behavior-linked indicators of attention (EG & CG)<br>Mental stillness and sustained attention (CG)<br>Neutral (CG)	113
Post	Teachers		Improved attention span (EG & CG)<br>Behavioral regulation supporting attention (EG & CG)	12

			Improved grasping (CG)<br>Neutral (EG)	
Interim	Students	Working memory	Improved academic retrieval and learning efficiency	05
Interim	Teachers		Neutral	01
Post	Students		Improved academic retrieval and learning efficiency (CG)<br>Improved daily recall and functional memory (CG)<br>Neutral (CG)	12
Post	Teachers		Enhanced Comprehension (EG)<br>Better memorization (EG)<br>Increased ability to recall (EG)<br>Neutral (CG)	07
EG: Experimental group; CG: Control group				

Supplementary table 7
Themes and subthemes of psychological well-being

Assessment Phase	Respondents	Themes	Subthemes	Frequencies
Interim	Students	Autonomy	Behavioral self-regulation and habit Control<br>Internalization of practice / Self-Initiated Discipline	05
Interim	Teachers		Student initiative & ownership<br>Academic Autonomy	02
Post	Students		Self-directed learning and academic autonomy (EG)<br>Social autonomy and thoughtful interpersonal behavior (CG)<br>Emotional autonomy and self-expression (CG)<br>Behavioral self-regulation & habit control (CG)	07

Post	Teachers		Self-initiated discipline (EG)<br>Behavioral autonomy (EG)<br>Neutral (CG)	04
Interim	Students	Positive relation	Expanded and strengthened friendships<br>Reduced conflicts<br>Improved communication patterns within peer or family contexts<br>Neutral	11
Interim	Teachers		Improved unity, mutual support, and more coordinated teamwork<br>Neutral	04
Post	Students		Greater empathy and ability to understand others' perspectives (EG&CG)<br>Improved communication patterns within peer or family contexts (EG&CG)<br>Expanded and strengthened friendships (CG)<br>Reduced conflict (EG & CG)	20
Post	Teachers		Improved unity, mutual support, and more coordinated teamwork (EG)<br>Neutral (EG & CG)	08
Interim	Students	Environmental mastery	Improved emotional regulation<br>Adaptive handling of interpersonal situations<br>Managing task-related and environmental demands<br>Neutral	15
Interim	Teachers		Reduced conflict frequency	01
Post	Students		Improved emotional regulation (EG & CG)<br>Adaptive handling of interpersonal situations (EG & CG)	13

			Managing task-related and environmental demands (CG)	
Post	Teachers		Neutral (EG)<br>Disciplinary intervention (EG)<br>Improved classroom management (CG)	07
Interim	Students	Personal growth	Improved emotional regulation (patience)<br>Improved academic confidence and participation	04
Interim	Teachers		Skill development<br>Cognitive growth, curiosity & initiative<br>Behavioral maturity & responsibility<br>Academic engagement<br>Behavioral adjustment<br>Neutral	12
Post	Students		Emotional regulation and behavioral maturity (EG)<br>Improved self-confidence (EG)<br>Creative expression and cognitive Growth (EG)<br>Academic improvement (CG)<br>Personal maturity (CG)<br>Social maturity & interpersonal growth (CG)	40
Post	Teachers		Skill development (CG)<br>Behavioral maturity & responsibility (CG)<br>Academic engagement (EG & CG)<br>Behavioral adjustment (EG & CG)<br>Neutral (EG)	12
Interim	Students	Self-acceptance	Enhanced emotional self-awareness and reflection<br>Improved acceptance of self and others	05

Interim	Teachers		Neutral	01
Post	Students		Improved acceptance of self and others (EG & CG)<br>Reduced irritability and greater emotional ease (EG & CG)<br>Enhanced emotional self-awareness and reflection<br>Neutral (EG)	17
Post	Teachers		Acceptance of evaluation and feedback (EG)<br>Ownership of actions and self-accountability (CG)	03
Interim	Students		Not applicable	00
Interim	Teachers	Purpose in life	Academic seriousness<br>Academic Interest & Intellectual Curiosity<br>Growing Academic Responsibility	04
Post	Students		Increased Academic Motivation & Positive Learning Orientation (EG & CG)<br>Goal-setting, Planning & Study Discipline (Purpose-Oriented)	09
Post	Teachers		Academic Interest & Intellectual Curiosity (EG & CG)<br>Neutral (CG)	04
EG: Experimental group; CG: Control group				

Supplementary table 8
Themes and sub-themes on dimensions of adjustment

Assessment phase	Respondents	Themes	Subthemes	Frequencies
Interim	Students	Adjustment to Self	Emotional Regulation & anger Management<br>Calmness / peace / freshness<br>Reduced anxiety and stress	11

			Reduced overthinking & mental quiet<br>Self-regulation in activities<br>Neutral	
Interim	Teachers		Improved emotional self-regulation	01
Post	Students		Emotional regulation and anger management (EG & CG)<br>Calmness / peace / freshness (EG & CG)<br>Reduced anxiety and stress (EG & CG)<br>Reduced overthinking & mental quiet (EG & CG)<br>Self-regulation in Activities (EG & CG)<br>Neutral (EG & CG)	68
Post	Teachers		Improved emotional self - regulation (EG & CG)<br>Neutral (CG)	04
Interim	Students	Adjustment to school	Improved classroom conduct, and reduced mischief<br>Improved emotional responses to school situations<br>Reduced fear and anxiety in school settings<br>Becoming calmer and more stable in performance contexts	10
Interim	Teachers		Improved discipline and adherence to norms<br>Persistent challenges among a small subset of students<br>Reduced roaming and disruptive behavior<br>Neutral	13
Post	Students		Reduced fear and anxiety in school settings (EG)<br>Improved classroom conduct, and reduced mischief (EG & CG)	05
Post	Teachers		Improved discipline and adherence to norms (EG & CG)<br>Persistent challenges among a small subset (EG & CG)	25

			Reduced roaming and disruptive behavior (EG & CG)<br>Neutral (EG & CG)	
Interim	Students	Adjustment to home	Cognitive reframing and improved self-acceptance within family comparisons<br>Improved emotional regulation<br>Reductions in conflict with siblings	12
Interim	Teachers		Not Applicable	00
Post	Students		Improved emotional regulation (EG & CG)<br>Improved conflict handling and reasoning (EG & CG)<br>Cognitive reframing and improved self-acceptance within family comparisons (CG)	12
Post	Teachers		Not applicable	00
Interim	Students	Adjustment to friends	Improved peer harmony and reduced conflict	01
Interim	Teachers		Not applicable	00
Post	Students		Constructive conflict resolution (EG)<br>Improved peer harmony and reduced conflict (CG)	05
Post	Teachers		Neutral (EG)<br>Peer aggression (CG)<br>Partial improvement in peer conflict (CG)	03
EG: Experimental group; CG: Control group				
